# UNC-16/JIP3 regulates early events in synaptic vesicle protein trafficking via LRK-1/LRRK2 and AP complexes

**DOI:** 10.1371/journal.pgen.1007100

**Published:** 2017-11-16

**Authors:** Bikash Choudhary, Madhushree Kamak, Neena Ratnakaran, Jitendra Kumar, Anjali Awasthi, Chun Li, Ken Nguyen, Kunihiro Matsumoto, Naoki Hisamoto, Sandhya P. Koushika

**Affiliations:** 1 National Centre for Biological Sciences-Tata Institute of Fundamental Research, Bangalore, Karnataka, India; 2 Department of Biological Sciences, Tata Institute of Fundamental Research, Mumbai, Maharashtra, India; 3 Department of Biological Sciences, Birla Institute of Technology and Science, Pilani, Rajasthan, India; 4 Group of Signaling Mechanisms, Nagoya University, Nagoya, Japan; 5 Center for *C*. *elegans* Anatomy, Albert Einstein College of Medicine, New York, New York, United States of America; Princeton, UNITED STATES

## Abstract

JIP3/UNC-16/dSYD is a MAPK-scaffolding protein with roles in protein trafficking. We show that it is present on the Golgi and is necessary for the polarized distribution of synaptic vesicle proteins (SVPs) and dendritic proteins in neurons. UNC-16 excludes Golgi enzymes from SVP transport carriers and facilitates inclusion of specific SVPs into the same transport carrier. The SVP trafficking roles of UNC-16 are mediated through LRK-1, whose localization to the Golgi is reduced in *unc-16* animals. UNC-16, through LRK-1, also enables Golgi-localization of the μ-subunit of the AP-1 complex. AP1 regulates the size but not the composition of SVP transport carriers. Additionally, UNC-16 and LRK-1 through the AP-3 complex regulates the composition but not the size of the SVP transport carrier. These early biogenesis steps are essential for dependence on the synaptic vesicle motor, UNC-104 for axonal transport. Our results show that UNC-16 and its downstream effectors, LRK-1 and the AP complexes function at the Golgi and/or post-Golgi compartments to control early steps of SV biogenesis. The UNC-16 dependent steps of exclusion, inclusion and motor recruitment are critical for polarized distribution of neuronal cargo.

## Author summary

Synaptic vesicles (SVs) have a defined composition and size at the synapse. The multiple synaptic vesicle proteins (SVPs) found on these vesicle membranes are synthesized at and trafficked out of the cell body in distinct transport carriers. However, we do not yet understand how different SVPs are sorted and trafficked to the synapse. We show that UNC-16/JIP3 plays a critical role, in a series of essential steps, to ensure proper membrane composition and size of the ensuing SVP carrier exiting the cell body. These processes are “exclusion” of resident Golgi enzymes followed by the “inclusion” of synaptic vesicle proteins in the same transport carrier. Regulation of composition and size seems to occur independently of each other and depends on two distinct AP complexes acting downstream to LRK-1. Our study further indicates that the composition of the transport carrier formed is important for the recruitment of motors and consequently for the polarized localization of SVPs.

## Introduction

The secretory pathway in a cell involves the synthesis and trafficking of proteins through the ER-Golgi network and their subsequent targeting to different sub-cellular compartments. Generation of a defined transport carrier, with a characteristic protein and lipid composition, along the trafficking pathways is known to involve at least three steps (a-c, see below), several occurring at the trans-Golgi network (TGN). (a) Protein sorting where the secretory cargo is segregated away from Golgi resident proteins [1–4]. For example, segregation of different Regulated Secretory Proteins (RSP) such as POMC occurs via receptor-mediated sorting [5,6]. (b) A post-sorting step where clustered cargo undergoes budding and separation to form a vesicular compartment from the donor membrane. In part, the adaptor protein (AP) complexes regulate such steps by recognizing signal sequences on proteins and ensuring that they are sorted into appropriate compartments [7,8]. The AP-1 complex recruits proteins like Clathrin, which causes membrane deformation followed by budding and scission from the TGN and post-Golgi compartments [9,10]. (c) A third step is the recruitment of specific motors, dependent on the characteristic membrane composition, constituting proteins and/or lipids, of the newly formed cargo. For example, AP-1 interacts with the Kinesin-3 motor KIF13A to coordinate endosomal sorting during melanosome biogenesis [11]. These events ensure the formation of a defined transport carrier that gets targeted to a specific sub-cellular compartment. Protein sorting occurs at post-Golgi compartments as well. For example, during the multi-step maturation of secretory granules, sorting of proteins also occur post-Golgi at intermediate compartments known as the immature secretory granules (ISGs) [12]. However, the genes that regulate such processes remain to be well understood.

Synaptic vesicle proteins (SVPs) are essential for neurotransmission and synaptic vesicles at the synapse are known to have a defined composition [13]. Several SVPs have transmembrane domains and are trafficked out of the TGN through the regulated secretory pathway to the synapse. Each synaptic vesicle found at the pre-synaptic bouton has an assortment of proteins important for processes such as neurotransmitter filling (VGLUT1 [14]), docking and neurotransmitter release (SNB-1, SNT-1 [15–17], and fusion of synaptic vesicles with the plasma membrane (UNC-13 [18]). It is not clear whether all of these proteins are found on a single SVP transport carrier as it exits the cell body. An earlier study indicates that different SVPs, for example Synaptophysin and SV2 are associated with distinct pools of membranous organelles [19]. This could imply that different SVPs might travel in separate compartments before they come together in a mature synaptic vesicle that is found at the synapse. However, another study suggests that most or all SVPs, including Synaptobrevin and SV2, are transported in a single transport carrier to the presynaptic active zone [20]. Studies using PC12 cell lines and *in vivo* studies in *C*. *elegans* have shown that the AP-3 complex is required for synaptic vesicle biogenesis, potentially directly from the Golgi, and for the axonal targeting of SVPs respectively [21–23]. SVPs are also known to require the molecular motor KIF1A for their exit from the cell body and transport to the synapse in multiple systems [24,25]. Thus, although proteins such as AP-3 and KIF1A have been implicated in SVP transport carrier biogenesis and trafficking, several aspects of these early steps remain unclear. For example, it is not fully understood how the AP complexes confer specificity to the sorting of different SVPs. Mechanisms that ensure separation of SVPs and/or lipids of the cargo membrane from those integral to the Golgi membrane is also poorly understood. Further, we do not know in what order these multiple steps are carried out during SVP trafficking and transport carrier biogenesis

JIP3/UNC-16/dSYD, a JNK-signaling scaffold protein present at the Golgi in *Drosophila*, is thought to have roles in protein trafficking [26–28]. The *C*. *elegans unc-16* mutants also show mis-trafficking and mis-accumulation of multiple neuronal cargo such as SVPs, dendritic receptors, lysosomes and early endosomes [28,29]. It is unclear how UNC-16, a molecule known to interact with and scaffold multiple kinases, including MAPK family members, carries out its trafficking roles. Among the UNC-16-interacting proteins, LRK-1, the *C*. *elegans* homolog of LRRK2, has been previously implicated in the polarized trafficking of SVPs and is also found at the Golgi, like UNC-16 [30,31]. Thus, UNC-16 and LRK-1 could play roles in the early steps of SVP protein trafficking.

In this study, we show that the SVP transport carriers formed in *unc-16* and *lrk-1* mutants have an altered composition and size. These mutants also show defects in polarized distribution of SVPs, which mis-localize to the dendrites [29,30]. We show that UNC-16 regulates the composition via LRK-1 by excluding Golgi enzymes from the SVP transport carrier and by increasing the incidence of co-transport of SNB-1 and RAB-3 in the same transport carrier. We show that size of the SVP transport carriers formed is determined by UNC-16 via LRK-1-dependent localization of the μ-component (UNC-101) of the AP-1 complex at the Golgi. We also show that the UNC-16 through LRK-1 and the AP-3 complex ensures that specific SVPs are included in the same transport carrier. Thus, based on genetic and biochemical evidence, we propose a novel role of UNC-16 in synaptic vesicle protein trafficking wherein it functions via LRK-1 to regulate exclusion and inclusion of proteins and consequently motor recruitment on the carrier. The AP-1 and AP-3 complexes likely function at the Golgi and/or post-Golgi intermediate compartments downstream to UNC-16 and LRK-1, to regulate respectively the size and composition of the SVP transport carrier formed in the neuronal cell body.

## Results

### Polarized distribution of proteins is lost in *unc-16* neurons

**Fig 1:**
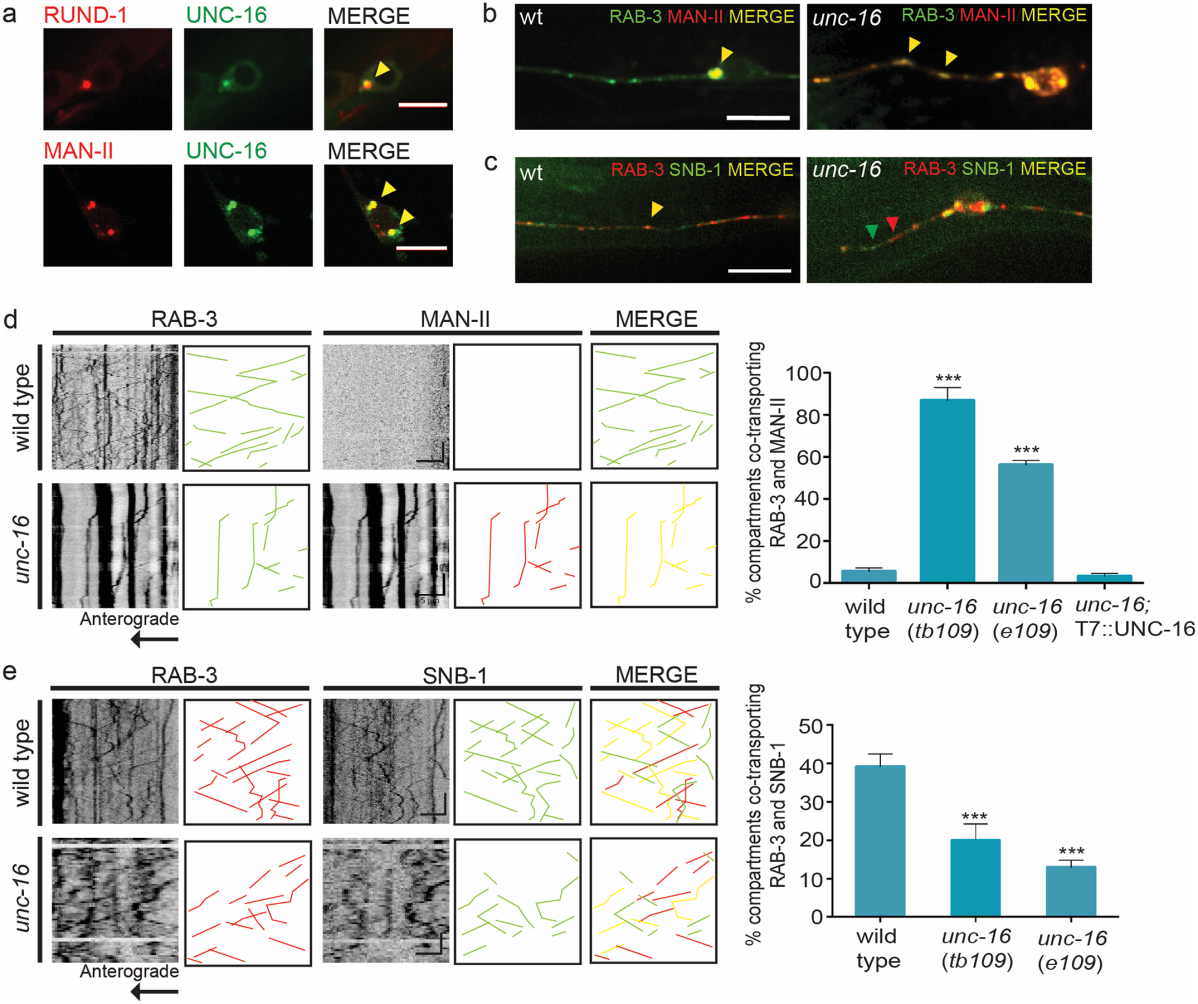
UNC-16 is essential to exclude Golgi enzymes from SVP transport carriers and to include certain SVPs in the same transport carrier. **(a)** UNC-16::GFP co-localizes with Golgi markers RUND-1::tagRFP and Mannosidase-II::mCherry and can be observed as 1–3 puncta in the cell bodies of PLM neurons. **(b)** and **(d)** Dual colour imaging and quantitation from kymograph analysis shows Golgi enzyme Man-II::mCherry is co-transported in the same compartment along with synaptic vesicle marker GFP::RAB-3 into the PLM neuronal processes in multiple *unc-16* alleles (*tb109* and *e109*). This mis-localization can be rescued by transgenic expression of wild type UNC-16 [*kmEx1000* (T7::UNC-16)]. Yellow arrowheads indicate co-localization of both GFP::RAB-3 and MAN-II::mCherry. **(c)** and **(e)** Dual colour imaging and quantitation from kymograph analysis shows the decreased incidence of synaptic vesicle proteins mCherry::RAB-3 and SNB-1::GFP travelling in the same compartment along the PLM neuronal process in multiple *unc-16* alleles (*tb109* and *e109*). Yellow arrowheads indicate co-localization of both mCherry::RAB-3 and SNB-1::GFP while red and green arrowheads represent either marker respectively. n ≥ 10 animals for wild type, *tb109* and *tb109; kmEx1000* and n = 6 for *e109* genotypes. Scale Bar: In kymographs, horizontal scale bar represents 5μm and vertical scale bar represent 40sec. In image panel, scale bar represents 10μm.

UNC-16/JIP3/dSYD has been implicated in the trafficking of multiple proteins such as those associated with synaptic vesicles and lysosomes [28,29,32]. To investigate the role it plays in regulating protein trafficking, we first examined the localization of UNC-16. Using two independent Golgi markers, Mannosidase-II (Man-II) and RUND-1 [33], we show that UNC-16 localizes to the Golgi in *C*. *elegans* neuronal cell bodies (Fig 1a). A similar localization of mammalian dSYD/JIP3 has been reported in CV-1 epithelial cells [26]. We isolated a new allele of *unc-16* (*tb109*), with an early stop codon at amino acid 423, that caused mis-trafficking of the trans-membrane VAMP Synaptobrevin-1 (SNB-1) to the dendrite of the amphid sensory neurons (Methods, S1a and S1b Fig, S1 Table). This phenotype is identical to those reported in other *unc-16* alleles [29]. The dendritic mis-localization of SNB-1 in *tb109* was rescued by the transgenic expression of wild type UNC-16 (S1b Fig, S1 Table). Upon examination of a dendritic receptor ODR-10 in the AWB neuron we found that, unlike in wild type animals, ODR-10 is ectopically localized to the axonal compartment in *unc-16* mutants (S1c Fig).

Loss of UNC-16 thus leads to the loss of polarized distribution of cargo in neurons. This mis-trafficking is not dependent on the orientation of microtubules, which is similar in both *unc-16* and wild type (S1d Fig). The defects in axonal and dendritic targeting along with the reported trafficking defects in dendritic, endosomal and lysosomal proteins suggests that UNC-16 acts as a general regulator of early events in the trafficking pathway [27–29,34].

### UNC-16 excludes Golgi enzymes from the SVP transport carriers

To understand the nature of early defects in *unc-16*, potentially occurring at the Golgi, we examined both—the localization of Golgi enzymes and whether cargo such as SVP transport carriers had altered composition.

Unlike wild type, in *unc-16* mutants, the Man-II enzyme mis-localizes to dendritic tips of the ASI neuron (S2g Fig, S1 Table) and both Man-II and Sialyl transferase (ST) are present as discrete compartments throughout the touch receptor neuron (TRN) process, up to the synapse (Fig 1b, S2b Fig), similar to reported observations [28,34]. Comparable to wild type, in *unc-16* mutants Golgi resident enzyme Man-II and ST continue to localize as 1–3 large puncta in the cell body (Fig 1b, S2b and S2d Fig). Other Golgi markers, such as RUND-1 and RAB-6.2, show a punctate distribution in the neuronal cell body of *unc-16* mutants, very similar to those observed in wild type animals, with no gross changes in the number or position of Golgi puncta (S2d Fig). This suggests that potentially only a subset of Golgi markers is mis-trafficked into the axons of *unc-16* animals.

In order to check if the mis-trafficking of Golgi proteins seen in *unc-16* alters SVP transport carriers, we carried out dual colour imaging of both Man-II and RAB-3. About 86% of RAB-3 containing compartments emerging from the cell body carry the Golgi enzyme Man-II, unlike in wild type where only ~ 5% of RAB-3 marked compartments co-transport Man-II (Fig 1d, S1 and S2 Movies). Similar observations were also made with the Golgi enzyme ST (S2a Fig). The mis-trafficking of Man-II along with RAB-3 into the neuronal process in *unc-16* mutants is rescued by the transgenic expression of wild type UNC-16 (Fig 1d).

Thus, UNC-16 is necessary to restrict Golgi resident proteins to the cell body and to exclude them from SVP transport carriers. The defects seen in *unc-16* are likely due to disruption of retention/retrieval mechanisms, leading to mis-trafficking of Golgi enzymes.

### UNC-16 regulates the composition and size of SVP transport carriers

**Fig 2:**
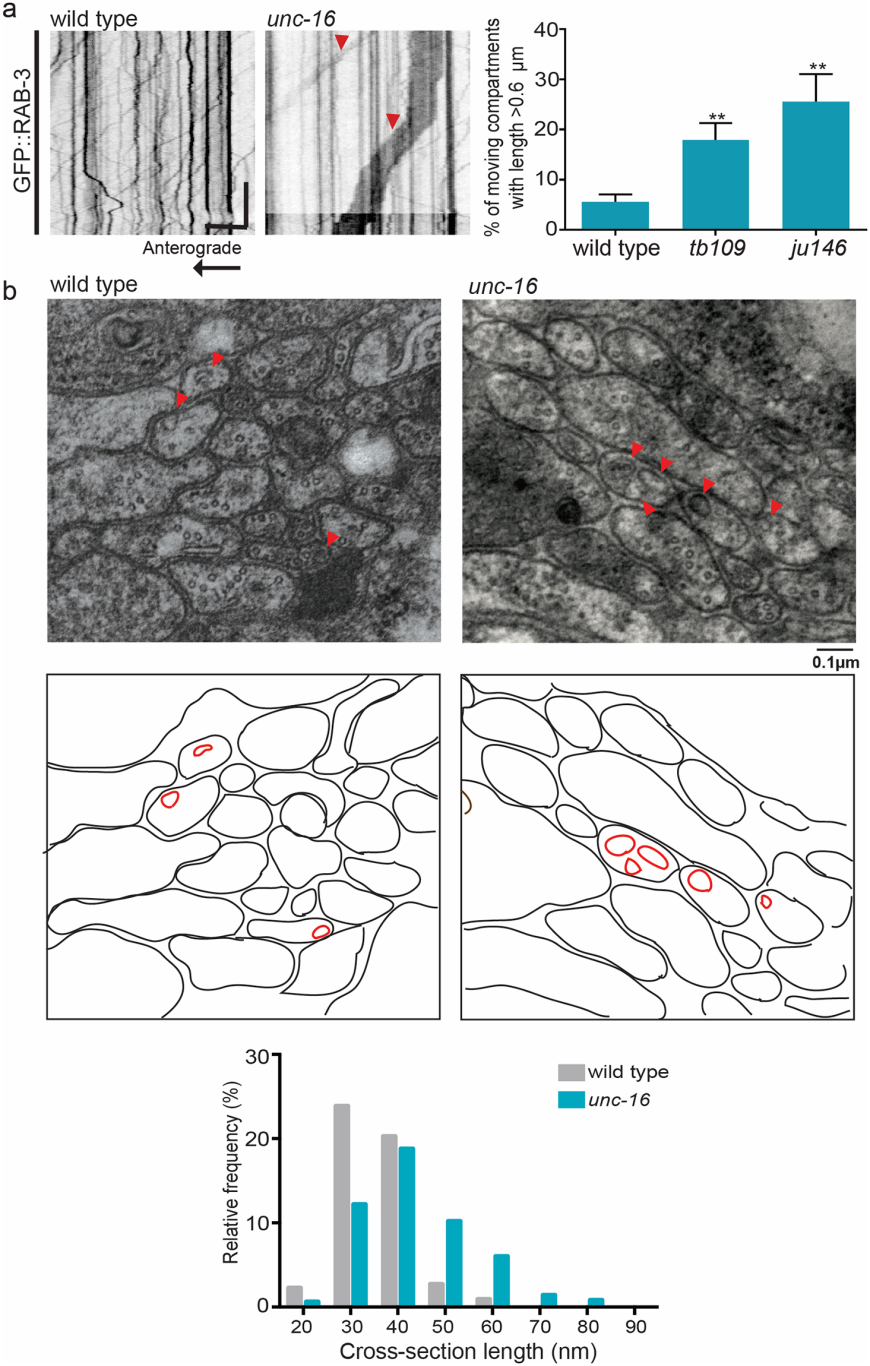
UNC-16 regulates the size of SVP transport carriers. **(a)** Kymograph analysis and quantitation of GFP::RAB-3 in PLM neuron shows increased presence of long moving compartments (indicated by red arrowheads) in *unc-16* (*tb109*) and *unc-16* (*ju146*) animals. n ≥ 10 animals, particles measured > 300 compartments per genotype **(b)** Electron micrographs of ventral cord section, representing non-synaptic regions, shows the presence of vesicular structures (indicated by arrowheads). A diagrammatic representation of the section is shown below the EM images to outline the large vesicular structures seen in *unc-16* (*tb109*) mutants compared to wild type animals. n = 5 animals for wild type and 9 animals for *tb109*. Scale Bar: In kymographs, the horizontal scale bar represents 5μm and vertical scale bar represent 40 sec. In electron micrograph scale bar represents 0.1μm.

To assess the membrane composition of the atypical SVP transport carriers formed in *unc-16*, we examined the co-transport of two synaptic vesicle proteins, RAB-3 and SNB-1, in TRNs. In wild type animals, the incidence of co-transport of RAB-3 and SNB-1 is ~ 39% of the mobile SVP transport carriers. In *unc-16* however, the frequency of co-transport of RAB-3 and SNB-1 reduces by half to ~19% (Fig 1c and 1e). This was corroborated by the reduction in co-localization of endogenous transmembrane Synaptotagmin (SNT-1) and RAB-3 at non-synaptic regions of the sub-lateral cord from ~95% in wild type to ~60% in *unc-16* animals (S1e Fig). Unlike in wild type, these erroneous SVP transport carriers exit the cell body as long tubular compartments. These long compartments show a ~1.5-fold increase in average length compared to those found in wild type and are three times more frequent in multiple alleles of *unc-16* across several neuronal cell types (Fig 2a, S3a Fig, S2 Table, S3–S5 Movies). Electron micrograph analyses showed an increase in the width of vesicles in non-synaptic regions of the dorsal and ventral nerve cord in *unc-16* compared to wild type animals (Fig 2b). Such larger vesicular profiles have been previously seen at the synapses of *unc-16* animals [34], [35]. The longer SVP transport carriers we see in our live imaging could contribute to these wider vesicular profiles observed in our electron micrographs (S4 Movie). We also verified that mutants in known interactors of UNC-16 such as *jnk-1*, *unc-116* and *dhc-1* [29,32,36] do not show these large moving tubular profiles carrying either SNB-1 or RAB-3, nor do they mis-localize the Golgi enzyme ST into the TRNs (S2 Table, S2b Fig).

Thus, UNC-16 facilitates “exclusion” of Golgi enzymes (see above) from SVP transport carriers, “inclusion” of specific SVPs into the same transport carrier and regulates the size of such compartments exiting the cell body.

### *lrk-1* mutants show a subset of the trafficking defects seen in *unc-16*

To identify other genes in the UNC-16-mediated SVP trafficking pathway, we examined mutants in LRRK2/LRK-1, known to be present on the Golgi [30]. LRRK2/LRK-1 regulates the trafficking and distribution of synaptic vesicles in presynaptic boutons [37] and *lrk-1* mutants show mis-trafficking of SNB-1 into dendrites of *C*. *elegans* chemosensory neurons, similar to phenotypes seen in *unc-16* animals [30].

**Fig 3:**
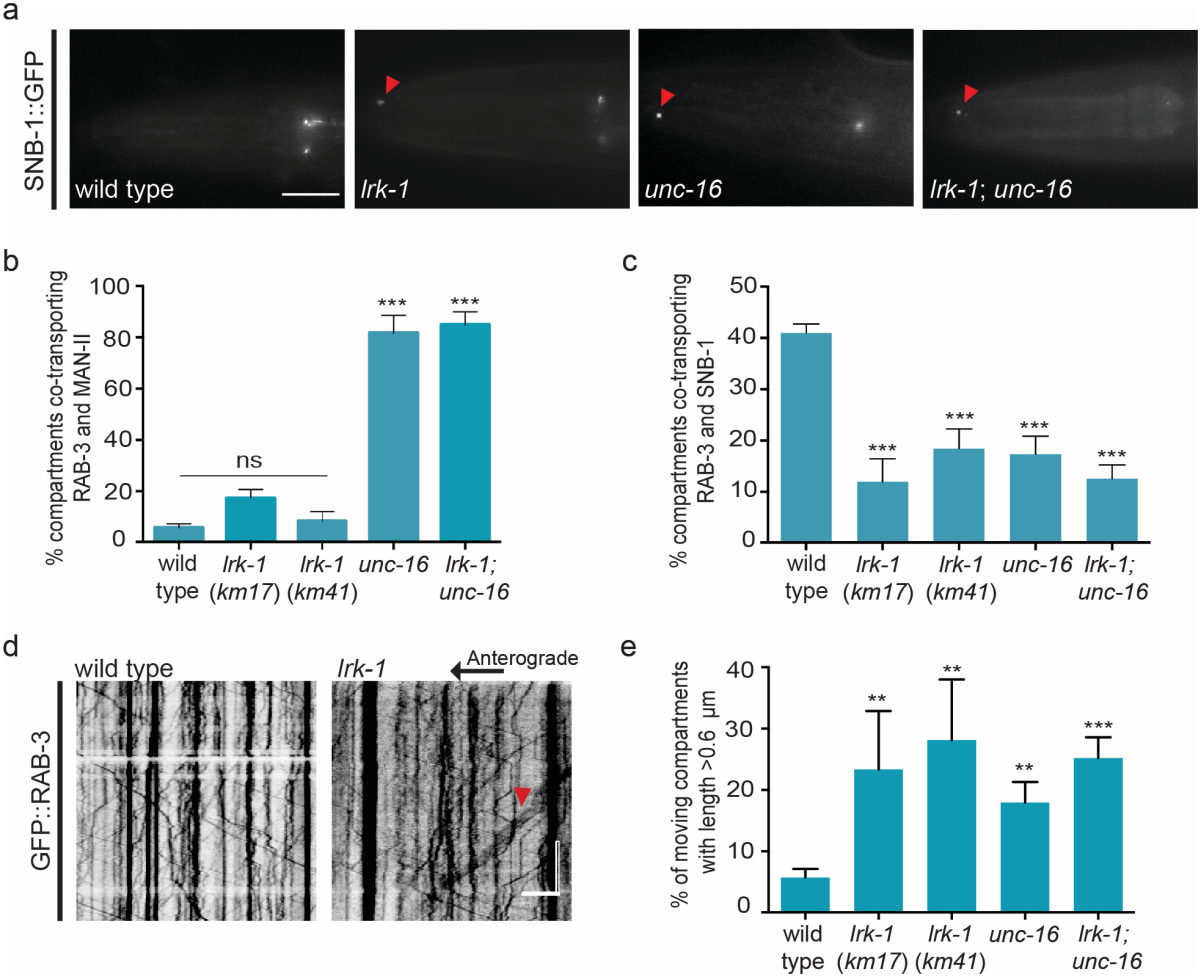
*lrk-1* mutants show a subset of mis-trafficking defects that is observed in *unc-16* mutants. **(a)** SNB-1::GFP is mis-localized into the dendritic compartment of the amphid sensory neuron in *lrk-1*(*km17*) and *lrk-1*(*km17*); *unc-16* (*tb109*) animals, similar to *unc-16* (*tb109*) animals. **(b)** Dual colour imaging and quantitation from kymograph analysis shows that Golgi enzyme Man-II::mCherry is not co-transported in the same compartment along with synaptic vesicle marker GFP::RAB-3 in the PLM neuronal processes of multiple alleles of *lrk-1* (*km17* and *km41*), unlike in *unc-16* (*tb109*) and *lrk-1*(*km17*); *unc-16*(*tb109*) animals. n ≥ 10 animals per genotype. **(c)** Dual colour imaging and quantitation from kymograph analysis shows that synaptic vesicle proteins mCherry::RAB-3 and SNB-1::GFP mostly travel in different compartments along the PLM neuronal process in multiple alleles of *lrk-1* (*km17* and *km41*), *unc-16* (*tb109*) and *lrk-1*(*km17*); *unc-16*(*tb109*) animals. n ≥ 10 animals per genotype **(d)** and **(e)** Kymograph analysis and quantitation of GFP::RAB-3 in PLM neuron shows increased presence of long moving compartments (indicated by red arrowheads) in multiple alleles of *lrk-1* (*km17* and *km41*) and *lrk-1*(*km17*); *unc-16*(*tb109*) double mutants. n ≥ 10 animals, particles measured > 300 compartments per genotype. Scale Bar: In kymographs, the horizontal scale bar represents 5μm and vertical scale bar represent 40 sec. In image panel, scale bar represents 10μm.

In *lrk-1* animals, Golgi enzymes Man-II and ST occasionally exit the cell body (Fig 3b and S2a Fig). However, unlike in *unc-16*, Man-II does not mis-localize to the dendrite in *lrk-1* animals (S1 Table, S2c Fig). In *lrk-1* animals the incidence of compartments carrying both Golgi enzyme and SVP is closer to wild type, with only ~18% of the mobile compartments co-transporting Man-II and RAB-3 (Fig 3b, S2a Fig). The incidence of co-transport of RAB-3 and SNB-1 in the same compartment reduces in *lrk-1* animals, with the severity of the phenotype similar to that observed in *unc-16* (Fig 3c). Additionally, there is a ~5-fold increase in the frequency of longer SVP transport compartments seen exiting the cell body in both alleles of *lrk-1* examined (Fig 3d and 3e, S3b Fig). As reported earlier, we also found that the dendritic marker ODR-10 shows a wild type-like polarized distribution in *lrk-1* animals [32].

Taken together, our observations suggest that LRK-1 plays a critical role in regulating the composition as well as the size of the SVP transport carriers formed at the cell body, potentially at the Golgi. However, *lrk-1* mutants lack the aberrant distribution of dendritic and Golgi proteins seen in *unc-16*.

### LRK-1 is necessary for UNC-16-mediated SVP trafficking

Since *lrk-1* has trafficking defects that are similar to *unc-16*, we tested whether UNC-16 and LRK-1 genetically function in the same pathway. We built double mutants and firstly assessed phenotypes present in *unc-16* but absent in *lrk-1*. The Golgi enzyme Man-II is mis-trafficked into RAB-3-containing compartments similar to *unc-16* single mutants (Fig 3b). Thus, the *lrk-1; unc-16* double mutants are similar to *unc-16* single mutants alone. Further, comparable to *unc-16* and *lrk-1* single mutants, the *lrk-1*; *unc-16* double mutants show loss of polarized distribution of SNB-1 (Fig 3a), reduced co-transport of RAB-3 and SNB-1 in the TRN process (Fig 3c) and an increased frequency of long moving compartments carrying RAB-3 (Fig 3e).

**Fig 4:**
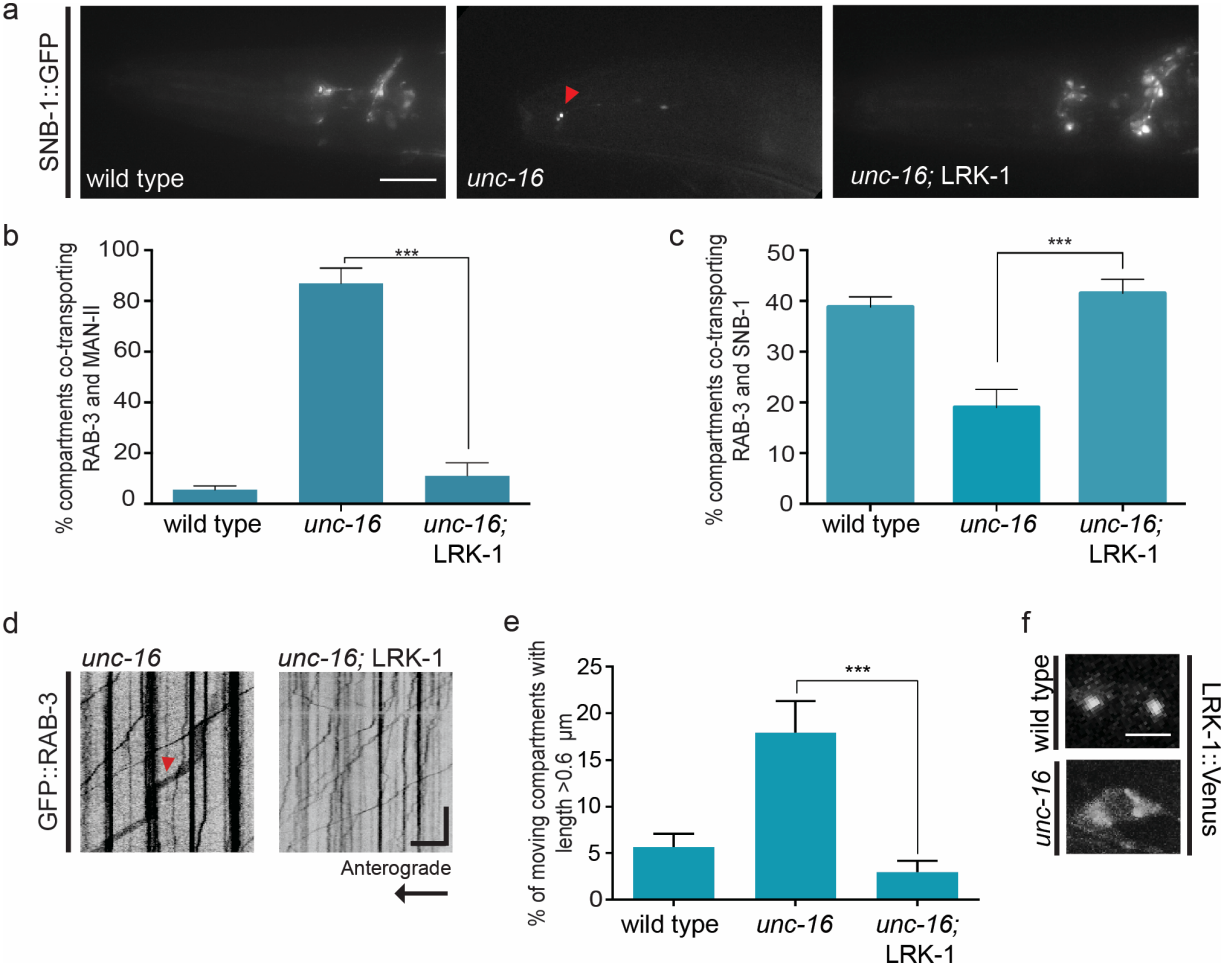
Overexpression of LRK-1 suppresses the SVP-related trafficking defects seen in *unc-16*. **(a)** SNB-1::GFP is mis-localized into the dendritic compartment of the amphid sensory neurons in *unc-16* (*tb109*) with the defect being suppressed in *unc-16* (*tb109*); *kmEx1180* (*LRK-1*::*FLAG*) animals. **(b)** Dual colour imaging and quantitation from kymograph analysis shows that Golgi enzyme Man-II::mCherry is not co-transported in the same compartment along with synaptic vesicle marker GFP::RAB-3 into the PLM neuronal processes of *unc-16; kmEx1180* animals. n ≥ 10 animals **(c)** Dual colour imaging and quantitation from kymograph analysis shows that synaptic vesicle proteins mCherry::RAB-3 and SNB-1::GFP predominantly travel in the same compartment along the PLM neuronal process in *unc-16* (*tb109*); *tbIs259* (*LRK-1*::*FLAG*) animals. n ≥ 10 animals **(d)** and **(e)** Kymograph analysis and quantitation of GFP::RAB-3 in PLM neuron shows reduced presence of long moving compartments in *unc-16; kmEx1180* animals. n ≥ 10 animals, particles measured > 200 per genotype **(f)** LRK-1::VENUS localization is altered in neuronal cell bodies of *unc-16* (*tb109*) mutants suggesting that UNC-16 regulates Golgi-localization of LRK-1. n ≥ 10 animals. Scale Bar: In kymographs, the horizontal scale bar represents 5μm and vertical scale bar represent 40 sec. In image panel, scale bar represents 10μm.

We next determined whether UNC-16 acts upstream of LRK-1 in the trafficking of SVPs by overexpressing transgenic LRK-1 in *unc-16* animals. Overexpression of LRK-1 greatly reduces the dendritic mis-localization of SNB-1 in *unc-16* animals (Fig 4a, S2 Table). Over-expression of LRK-1 was also sufficient to exclude Golgi enzyme Man-II from RAB-3 containing SVP transport carriers (Fig 4b), to restore incidence of co-transport of RAB-3 and SNB-1 to wild type (Fig 4c) and to reduce the frequency of long transport carriers to frequencies seen in wild type (Fig 4d and 4e, S3c Fig). Interestingly, the overexpression of LRK-1 in *unc-16* animals does not suppress the mis-trafficking of the dendritic marker ODR-10 into the axon (S1c Fig) nor does it completely suppress the exit of Man-II into the neuronal process (although these no longer travel in RAB-3 containing transport carriers). This suggests that transgenic LRK-1 only ameliorates the SVP-specific trafficking defects observed in *unc-16*.

As LRK-1 and UNC-16 are both present on the Golgi, we examined the localization of each protein and found that in *unc-16* animals the punctate localization of LRK-1 on the Golgi is reduced in neuronal cell bodies (Fig 4f). In *unc-16*, a 10-fold increase was seen in the number of cell bodies showing a completely diffuse localization of LRK-1, compared to wild type animals (S4d Fig). In addition, LRK-1 is also mis-localized into the dendrites of sensory neurons in *unc-16* animals (S4c Fig). On the other hand, UNC-16 localization remains unaffected in *lrk-1* animals (S4e Fig). We further examined if both these proteins were part of the same complex. Immunoprecipitation experiments from *C*. *elegans* expressing LRK-1::FLAG and UNC-16::GFP show that LRK-1 and UNC-16 are present together in a complex *in vivo* (S4a and S4b Fig).

Given that overexpression of LRK-1 in *unc-16* is sufficient to restore the processes of exclusion, inclusion and size regulation, our data suggest that *unc-16* functions genetically upstream of *lrk-1*. This, along with the observation that *lrk-1* mutants have modest exclusion defects but inclusion defects similar in severity to *unc-16*, suggests that exclusion may precede inclusion during SV biogenesis. The presence of UNC-16 likely facilitates the localization of LRK-1 on the Golgi and both together, possibly in a complex, regulate the trafficking of multiple SVPs.

### The AP-1 complex regulates the size of the SVP transport carrier

Dendritic trafficking of proteins is known to require the AP-1 complex [10,23,38]. The mis-trafficking of SVPs into dendrites in *lrk-1* mutants is dependent on UNC-101, the μ-chain of the Adaptor protein-1 (AP-1) complex in *C*. *elegans* [30]. Therefore, we tested if UNC-101 is involved in the UNC-16 and LRK-1-mediated regulation of the composition and size of the SVP transport carrier.

**Fig 5:**
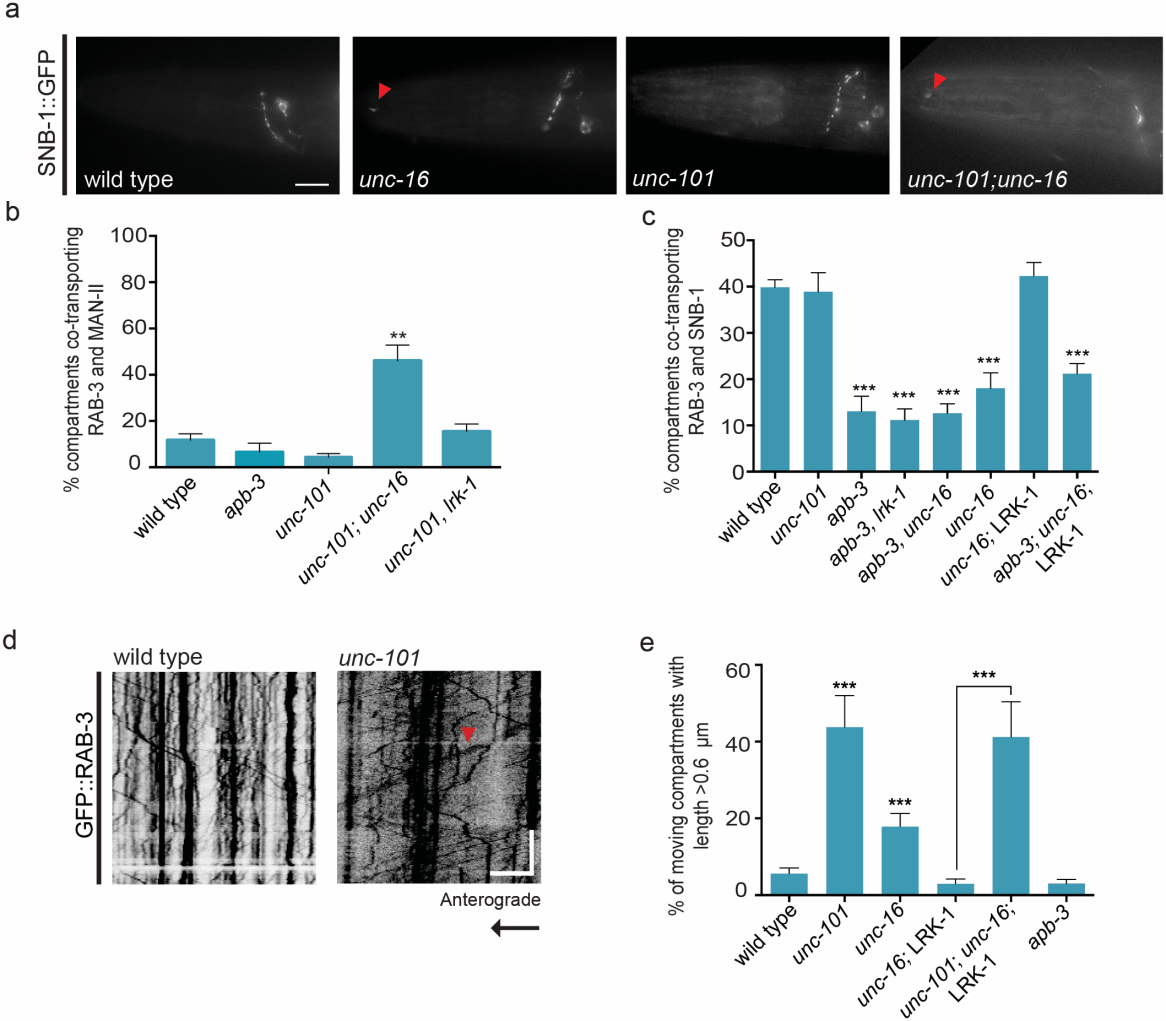
*unc-101* and *apb-3* acts downstream of *unc-16* and *lrk-1* to regulate size and composition of SVP transport carriers respectively. **(a)** SNB-1::GFP is mis-localized into the dendritic compartment of the amphid sensory neurons in *unc-16* (*tb109*) and *unc-101* (*m1*); *unc-16* (*tb109*) but not in wild type or *unc-101* (*m1*) animals **(b)** Dual colour imaging and quantitation from kymograph analysis shows Golgi enzyme Man-II::mCherry is co-transported with synaptic vesicle marker GFP::RAB-3 into the PLM neuronal process in *unc-101; unc-16* but not in *unc-101* (*m1*), *unc-101* (*m1*), *lrk-1* (*km17*) and *apb-3* (*ok429*) animals. n ≥ 10 animals **(c)** Dual colour imaging and quantitation from kymograph analysis shows co-transport of GFP::RAB-3 and SNB-1::GFP in the PLM neuronal process reduces in *apb-3* (*ok429*), *apb-3* (*ok429*), *lrk-1* (*km17*), *apb-3* (*ok429*); *unc-16* (*tb109*) and in *apb-3* (*ok429*); *unc-16* (*tb109*); *tbIs259* (*LRK-1*::*FLAG*) but not in *unc-16* (*tb109*); *tbIs259* (LRK-1::FLAG) or *unc-101* (*m1*) mutants. n ≥ 10 (*LRK-1::FLAG*) animals **(d)** and **(e)** Kymograph analysis and quantitation of GFP::RAB-3 in PLM neuron shows increased presence of long moving compartments (indicated by red arrowheads) in *unc-101* (*m1*) and in *unc-101* (*m1*); *unc-16* (*tb109*); *kmEX1180* (*LRK-1*::*FLAG*) animals but not in *apb-3* (*ok429*) animals. n ≥ 10 animals, particles measured > 200 compartments per genotype. Scale Bar: In kymographs, the horizontal scale bar represents 5μm and vertical scale bar represent 40 sec. In image panel, scale bar represents 10μm.

In *unc-101* animals, like in wild type TRNs, Man-II and ST are restricted to the cell body and the incidence of co-transport of SVPs RAB-3 and SNB-1 is about 40% (Fig 5b and 5c, S2a Fig). Thus UNC-101, unlike UNC-16 and LRK-1 does not appear to play a role in regulating the composition of SVP transport carriers. However, nearly 40% of the RAB-3 containing vesicles in *unc-101* animals were longer in the PLM neuron (Fig 5d and 5e, S3d Fig, S6 Movie), similar to *unc-16* (Fig 2a, S4 and S5 Movies) and *lrk-1* (Fig 3d and 3e) mutants. These longer transport carriers were seen in several other neuronal types (S7 Movie) as well. Thus, UNC-101 is involved in regulating the size of the SVP transport carrier leaving the cell body. In order to genetically position UNC-101 relative to UNC-16 and LRK-1, we built double mutants with both *unc-16* and *lrk-1*. The mis-trafficking of the Golgi enzyme Man-II in the double mutants *unc-101*; *unc-16* and *unc-101*; *lrk-1* animals were similar to *unc-16* or *lrk-1* single mutants respectively (Fig 5b). Further, the rescue of size defects in *unc-16* mutants by LRK-1 over-expression was found to require UNC-101 (Fig 5e, S3d Fig). Thus, UNC-101 acts downstream of both UNC-16 and LRK-1 in regulating size of the transport carrier but does not appear to influence sorting of SVPs into the carrier.

Previous studies have shown that the aberrant trafficking of SNB-1 into the dendrite of the sensory neuron in *lrk-1* animals is dependent on UNC-101 [30]. Consistent with this, we observed that in *unc-101*, *lrk-1* animals SNB-1 is excluded from the dendrite (S2 Table). In *unc-101; unc-16* double mutants, SNB-1 continues to be mis-localized to the dendrite in ~ 20% of the animals, unlike in *unc-16* animals where mis-localization is seen in 100% of the animals (Fig 5a, S2 Table). The MAN-II mis-localization into the dendrites in *unc-16* and *lrk-1* also requires UNC-101 (S2c Fig). Thus, even in absence of LRK-1 or UNC-16, UNC-101 continues to regulate the trafficking of proteins into dendrites. Our data, thus, suggests two roles for UNC-101 –(i) regulation of dendritic trafficking and (ii) regulating the size of axonally trafficked SVP transport carrier.

### The AP-3 complex regulates sorting of SVPs into a transport carrier

The AP-3 complex is known to function downstream to LRRK2 in the trafficking of lysosomal membrane proteins and to sort axonal proteins away from dendritic proteins [23,39]. Thus, we examined SVP trafficking in *apb-3* mutants defective in the AP-3 β-subunit.

Unlike *unc-101* animals, *apb-3* mutants do not have longer SVP transport carriers, suggesting that AP-3 is not involved in size regulation (Fig 5e). Further, similar to wild type and *unc-101* mutants, the Golgi protein Man-II largely stays restricted to the cell body in *apb-3* mutants with little or no co-transport of Man-II with RAB-3 (Fig 5b). However, the *apb-3* mutants show inclusion defects wherein incidence of co-transport of RAB-3 and SNB-1 is reduced to ~14% (Fig 5c), similar to *unc-16* or *lrk-1* single mutants. Both *apb-3*; *unc-16* and *apb-3*, *lrk-1* double mutants show reduced co-transport of RAB-3 and SNB-1, similar in severity to that seen in *unc-16* or *lrk-1* single mutants alone (Fig 5c). Additionally, over-expression of LRK-1 in *unc-16* mutants is unable to rescue the inclusion defects in absence of APB-3 (Fig 5c). Thus, the AP-3 complex acts downstream of LRK-1 and may have roles in UNC-16 and LRK-1-mediated regulation of composition of the SVP transport carrier formed.

### Golgi-localization of UNC-101 depends on LRK-1

**Fig 6:**
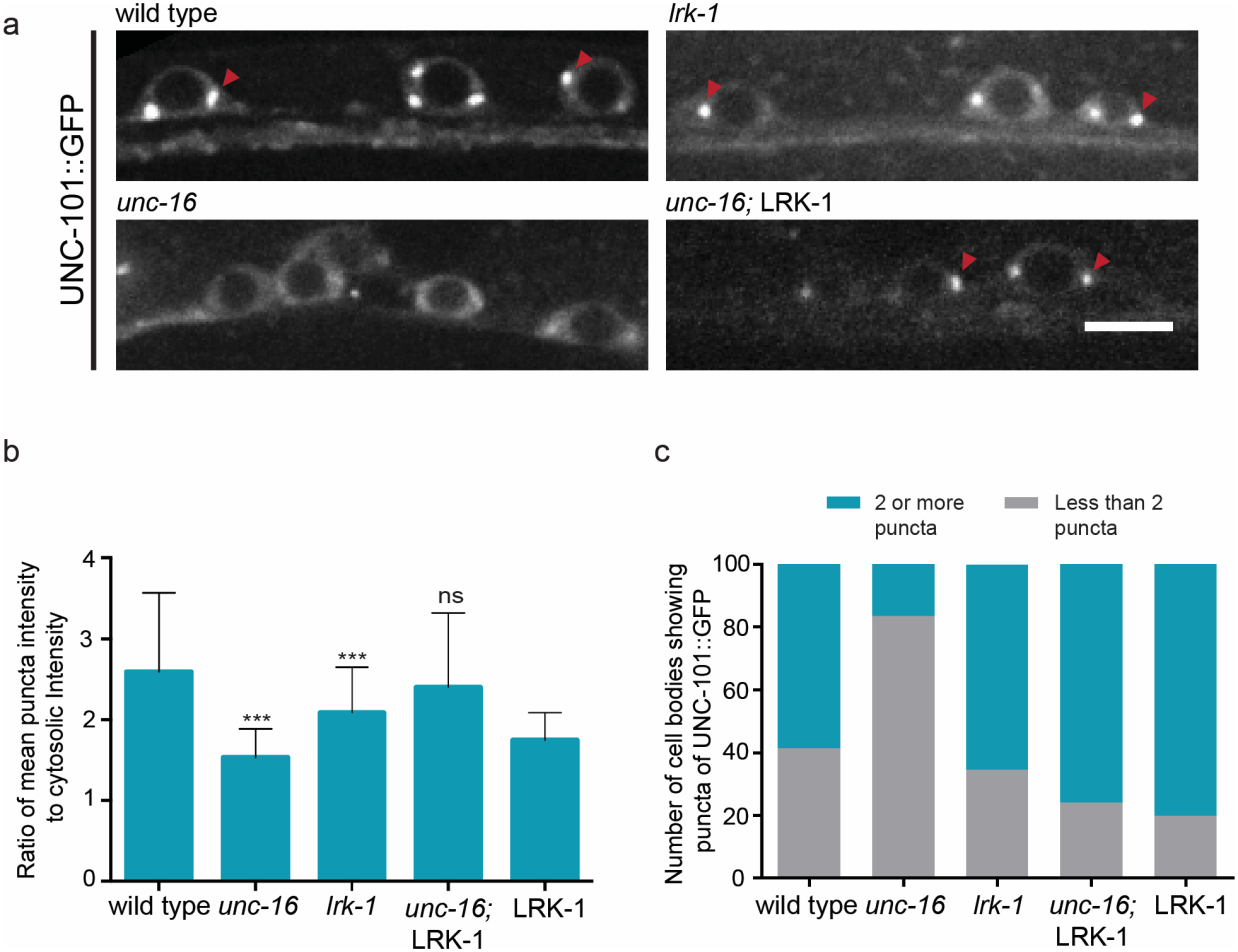
UNC-16 and LRK-1 regulate the localization of UNC-101 to the Golgi. **(a)** UNC-101::GFP shows reduced localization to the Golgi in the cell bodies of motor neurons in *unc-16* (*tb109*) and *lrk-1* (*km17*) animals but not in wild-type and *unc-16* (*tb109*); *kmEx1180* animals **(b)** Quantitation of fold change in UNC-101::GFP puncta shows reduced intensity in *unc-16* (*tb109*) and *lrk-1* (*km17*) animals **(c)** Quantitation of number of UNC-101::GFP puncta in different genotypes indicated. n ≥ 10 animals per genotype. Scale Bar represents 10μm.

As the phenotype of long moving compartments observed in *unc-16*, *lrk-1* and *unc-101* is similar, we investigated whether there were changes in reported Golgi localization of UNC-101 in *unc-16* and *lrk-1* [23]. UNC-101 is not present as a defined puncta in *unc-16* neuronal cell bodies while in *lrk-1* animals, the puncta are reduced in both number and intensity as compared to wild type (Fig 6a, 6b and 6c). The overexpression of transgenic LRK-1 in *unc-16* animals restores the localization of UNC-101 on the Golgi (Fig 6a, 6b and 6c). Since overexpression of LRK-1 rescues the size defects seen in *unc-16* but not in an *unc-101; unc-16* background, the LRK-1 mediated Golgi localization of UNC-101 is required for maintaining the size of the SVP transport carriers Figs 4e and 5e). Thus, the altered size of the synaptic vesicle precursors observed in *unc-16* and *lrk-1* depends on the presence of UNC-101 (or the AP-1 complex) on the Golgi.

### SVPs in *unc-16* are independent of UNC-104 motor

**Fig 7:**
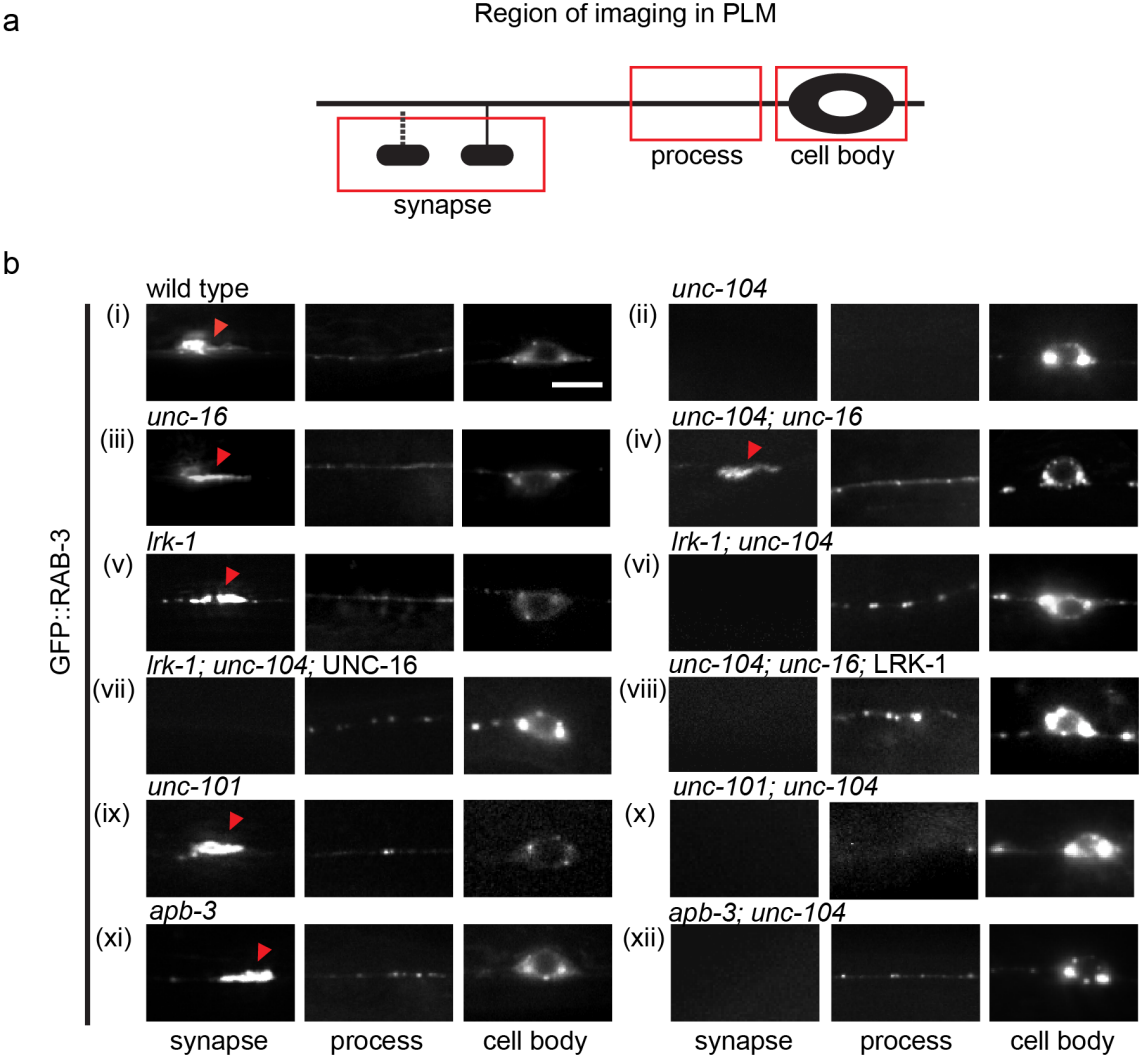
Genetic interactions between UNC-16, LRK-1 and UNC-104. **(a)** Schematic of PLM neuron with the region of imaging (cell body, process or synapse) marked within a red box **(b)** GFP::RAB-3 localization in the cell body, process and synapse of PLM of different genotypes (indicated above each image panel). n ≥ 10 animals per genotype. Scale Bar represents 10μm.

SVP transport carriers in *unc-16* are thought to recruit non-canonical motors [29,36], which may arise as a consequence of altered membrane composition. The molecular motor UNC-104 is known to transport synaptic vesicles protein transport carriers in *C*. *elegans* [24,39]. In concordance with this role, in *unc-104* mutants, the SVPs RAB-3 or SNB-1 are found to be trapped in the cell body and absent from synapses (Fig 7bii) [15,40–42]. A previous study has shown that SVPs formed in *unc-16* are transported independently of the UNC-104 motor in the DD and VD motor neurons [29]. This loss of motor-cargo specificity is also observed in TRN neurons such that SVP transport carriers travel up to the synapse in *unc-16; unc-104* animals (Fig 7biii and 7biv). In *lrk-1*; *unc-104* and in *apb-3*; *unc-104* double mutants RAB-3 was observed in the proximal portion of the neuronal process but not at synapses, suggesting that the SVP carriers formed in *lrk-1* and *apb-3* are only partially dependent on UNC-104 (Fig 7bv, 7bvi and 7bx). The RAB-3 containing vesicles in *unc-101; unc-104* were restricted to the cell body of the TRNs like in *unc-104* alone (Fig 7bix) suggesting that unlike in *unc-16* or *lrk-1* mutants, the SVP transport carriers in *unc-101* mutants were completely dependent on the UNC-104 motor.

We tested if the overexpression of LRK-1 was sufficient to restore the dependence of the SVP transport carrier on UNC-104 in *unc-16* mutants and found that in these animals the SVP transport carriers are partially dependent on UNC-104 and are unable to reach the synapse (Fig 7bviii). Conversely, transgenic expression of UNC-16 does not significantly affect the altered dependence of the SVP transport carrier on UNC-104 in *lrk-1* animals (Fig 7bvii). These observations demonstrate that the overexpression of LRK-1 in *unc-16* sufficiently changes the composition of the SVP transport carrier such that it is now largely dependent on UNC-104. This further suggests that composition of the SVP transport carrier is critical to allow sufficient UNC-104 motor recruitment or to potentially exclude other motors along with excluding other proteins.

## Discussion

The mechanisms by which synaptic vesicle proteins (SVPs) are sorted into transport carriers and trafficked out of the cell body are not yet clearly understood. It is thought that like other membranous cargo such as secretory granules and dense core vesicles, SVPs are sorted at the Golgi and post-Golgi compartments and the transport carrier that is formed moves out of the cell body by recruiting specific motors, such as KIF1A/UNC-104 [1,4,5,43–45]. Using the model system *C*. *elegans*, we have uncovered a novel role for UNC-16/JIP3 in the trafficking and biogenesis of SVP transport carriers. Given that UNC-16/JIP3 localizes to the Golgi in *C*. *elegans* (Fig 1a) as well as in mammalian epithelial cells [26], it likely has conserved functions at the Golgi. We show that the SVP transport carrier biogenesis roles of UNC-16 occur via LRK-1. The sorting roles of UNC-16 and LRK-1 lead to the polarized distribution of SVPs as well as regulation of its composition and size. These two proteins regulate the composition of SVP transport carriers via the exclusion of Golgi resident enzymes and inclusion of relevant SVPs. These roles depend on the AP complexes where the AP-1 complex regulates the size of the carrier and the AP-3 complex regulates composition.

Our study shows that UNC-16 is essential to prevent Golgi resident enzymes such as Mannosidase-II and Sialyl transferase from entering the SVP transport carrier as inferred from the observed mis-trafficking of these enzymes along the neuronal process in *unc-16* mutants (Fig 1b and 1d and S2a Fig). Previous studies have shown that retention of Golgi enzymes could occur through several mechanisms such as binding to scaffolding proteins on the Golgi or through the regulation of the lipid membrane composition to favour partitioning of different membrane proteins [46–48]. UNC-16 potentially acts as a scaffolding molecule to recruit effectors such as LRK-1 on the Golgi (Fig 4f), through which it may function to retain certain Golgi resident enzymes. Although *lrk-1* mutants themselves do not show exclusion defects (Fig 3b), an excess of LRK-1 in an *unc-16* background is sufficient to bypass the requirement of UNC-16 to exclude Golgi resident enzymes from the SVP transport carrier (Fig 4b). This suggests that LRK-1 may have redundant roles in exclusion of Golgi enzymes from SVP transport carriers. Alternatively, LRK-1 could act in an analogous manner to LRRK2, the mammalian homolog of LRK-1, that has been implicated previously in regulating the retromer complex, which sorts proteins from the endosome-lysosome degradation pathway retrogradely to the Golgi complex [49,50]. This likely occurs through the action of certain RABs such as RAB-7 and RAB-9 and through LRK-1’s interaction with VPS35 of the retromer complex [49–51]. In *unc-16* mutants Golgi-resident proteins may not be excluded from other compartments as well, such as lysosomes. Our data suggests that UNC-16 mediated LRK-1 localization facilitates the exclusion of Golgi enzymes specifically from the SVP transport carrier as Man-II continues to be ectopically present along the axon potentially in other compartments in *unc-16* mutants overexpressing LRK-1.

An important step during protein sorting is the clustering or segregation of proteins to be placed into the same compartment away from the donor compartment proteins. For example, the SVP Synaptobrevin-II and Synaptophysin interact with each other resulting in their co-trafficking [52,53]. Our findings suggest that both UNC-16 and LRK-1 are required for ensuring certain SVPs (such as SNB-1, SNT-1, and RAB-3) are sorted together and included more frequently in the same transport carrier (Figs 1c, 1e and 3c and S1e Fig). Moreover, the inclusion defects seen in *unc-16* can be rescued by overexpression of LRK-1 and this rescue depends on presence of a functional AP-3 complex (Figs 4c and 5c). Along with the observation that *lrk-1* genetically lies downstream to *unc-16* and that exclusion defects are seen only in the *unc-16* mutants, we hypothesize that exclusion of Golgi enzymes likely precedes inclusion during the early steps of SVP sorting. In *unc-16* animals, the localization of LRK-1 itself at the Golgi is disrupted (Fig 4f, S4d Fig) suggesting that *unc-16* may act as a potential hypomorph of LRK-1. This, along with our biochemical evidence that UNC-16 and LRK-1 are present in the same complex (S4a and S4b Fig), suggests that UNC-16 may scaffold LRK-1 at the Golgi in a physical complex that specifically regulates sorting of SVPs.

In addition to protein sorting, a crucial step in the formation of a transport carrier involves the regulation of its size. Previous studies have shown that proteins present on the surface of the TGN are crucial for the recruitment of the machinery involved in size regulation such as the Adaptor protein complexes [8,9,54–57]. We found that UNC-101, the μ-chain of the AP-1 complex in *C*. *elegans*, is indeed engaged in regulating the size of the SVP transport carrier formed (Fig 5d and 5e, S3d Fig). Importantly, the localization of UNC-101 on the Golgi is regulated by UNC-16 and LRK-1 (Fig 6). Considering that the Golgi enzyme Man-II in all of these mutants show the presence of 2–3 large puncta juxtaposed to the nucleus in the cell body, like in wild type, the Golgi is likely intact and the appearance of Golgi resident enzymes in the neuronal process can be accounted for due to errors in sorting/retrieval of these proteins. Furthermore, the size regulation by UNC-101 acts downstream to the early sorting steps regulated by UNC-16 and LRK-1 since overexpression of LRK-1 was able to rescue the size in *unc-16* animals but not in *unc-16*; *unc-101* mutants (Figs 4e and 5e, S3d Fig). Since exclusion or inclusion defects were absent from *unc-101* animals, this also suggests that regulation of size and membrane composition could be independent processes and having an unusually large size does not necessarily incorporate other proteins (Golgi enzymes) typically excluded from these carriers. Earlier studies have also shown that the AP-1 complex is required for trafficking of proteins into dendrites [10,23,38]. Consistent with these observations, the dendritic mis-localization of SVPs in our mutants was largely dependent on UNC-101 (Fig 5a, S2 Table). We report an additional role where it regulates the size of the axonal cargo viz. SVP transport carriers. A recent study showed that the AP-3 complex is necessary at the Golgi for axonal localization of proteins [23]. AP-3 has previously been implicated in the sorting of proteins from early endosomes to lysosomal compartments [58–60]. Our study suggests that, unlike AP-1, the AP-3 complex does not regulate the size of SVP transport carriers (Fig 5e). On the other hand, compared to wild type animals, the *apb-3* mutants show inclusion defects wherein the co-transport of SVPs, SNB-1 and RAB-3, is reduced (Fig 5c), which is similar to but more severe than that observed in *unc-16* mutants. A recent study showed that AP-3 complex acts downstream of LRK-1 in the endo-lysosomal trafficking pathway [61]. Since overexpression of LRK-1 is able to suppress the defects in *unc-16* but not in an *apb-3; unc-16* double mutant (Fig 5c), AP-3 likely functions downstream to both UNC-16 and LRK-1 in regulating composition of some SVP transport carriers. Our data suggests that LRK-1 may be a general means to recruit different AP complexes to the appropriate membrane surface. We further hypothesize that UNC-101 at the Golgi is required for formation of an intermediate compartment whose size itself may be regulated by the AP-1 complex. At such an intermediate compartment the AP-3 complex may function to regulate the composition but not the size of vesicles arising from this precursor. Alternatively, UNC-101 may also have an additional role at the intermediate compartment to regulate size of vesicles arising from this compartment. LRK-1, itself may exert its retromer function at such an intermediate compartment for retrieval of Golgi enzymes.

**Fig 8:**
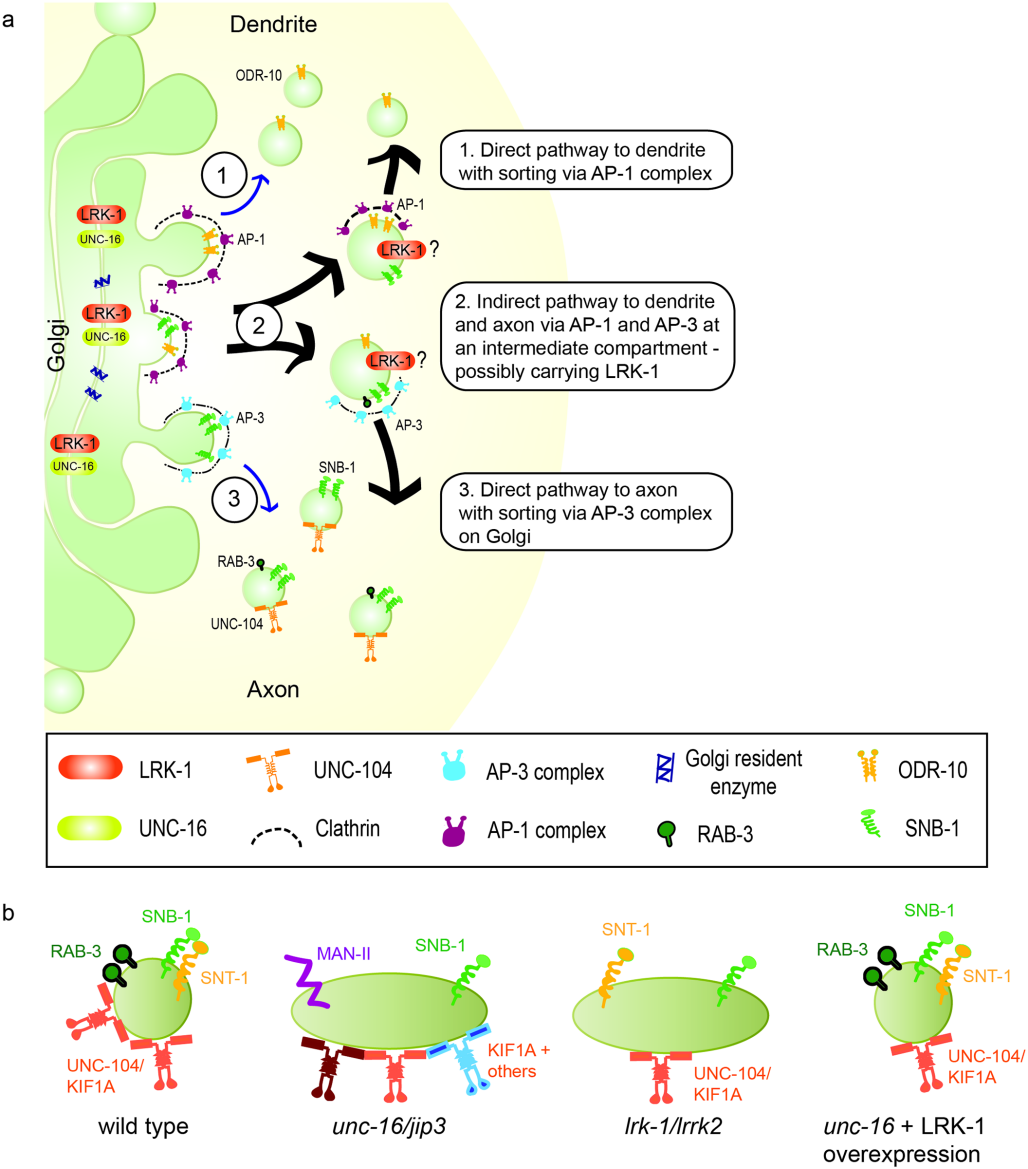
UNC-16, via LRK-1 and the AP complexes, functions at Golgi and/or post-Golgi intermediate compartments to regulate composition and size of SVP transport carriers. **(a)** UNC-16 and LRK-1 are present in a physical complex at the Golgi (possibly also at post-Golgi compartments) where it functions to regulate the composition of the SVP transport carriers formed. This involves at least two processes–(i) “exclusion” of Golgi enzymes from the SVP transport carrier whereby Golgi enzymes such as Mannosidase-II are retained at the Golgi and (ii) “inclusion” of different SVPs into the same transport carrier. The two AP complexes—namely AP-1 and AP-3 –are known to function at the Golgi to regulate dendritic and axonal trafficking of proteins respectively [indicated by (1) and (2)]. In addition, we propose that the two AP complexes, downstream to LRK-1, may act post-Golgi through an intermediate compartment [indicated by thick arrows (3)]. The AP-1 complex regulates the size of the SVP transport carriers emerging from these intermediate compartments, whose size itself may also be regulated by AP-1. The AP-3 complex functions to regulate the compoisition of the emerging SVP transport carriers. Further, such an intermediate compartment may also carry LRK-1 that is involved in the retrieval of Golgi proteins via the retromer complex. **(b)** schematic representation of the SVP transport carriers formed in different genotypes.

Based on all our observations, the SVP transport carriers formed in *unc-16* seem to be visibly aberrant in nature (Fig 8b). These observed defects likely arising due to an altered surface composition might also lead to the recruitment of multiple motors as suggested by Byrd *et al*., 2001 [29]. Consistent with this, we observed that the aberrant SVP transport carriers formed in *unc-16* were no longer dependent exclusively on UNC-104 (Fig 7b). Further, it also appears to depend at least partially on other motors such as UNC-116/Kinesin-1 ([29]; S4f Fig). This suggests that the motor-cargo specificity is lost in *unc-16* perhaps as a consequence of the altered surface composition that might now contain adaptors for multiple other motors. The transport carriers formed in *lrk-1* and *apb-3*, have an altered composition, as suggested by defects in the ability to include different SVPs, and are only partially dependent on UNC-104 (Fig 7bvi and 7bx). This could again imply that due to an altered composition the carriers in *lrk-1* and *apb-3* mutants are not able to stably recruit sufficient numbers of the SV motor. Over expression of LRK-1 in *unc-16* completely restores co-transport of RAB-3 and SNB-1, but is still insufficient to make the SVP transport carriers completely dependent on the motor (Fig 7bviii) suggesting the existence of additional factors necessary for forming an UNC-104 motor dependent transport carrier.

Previously, UNC-16 has been suggested to have a “clearance function” wherein it regulates the retrieval of cell soma organelles such as lysosomes and endosomes from the axon, thereby acting as an “organelle gatekeeper” [27,28]. Considering the multiplicity of mis-sorting defects we see in *unc-16*, we postulate that the unusual accumulation of different organelle proteins in the axon of these mutants could also be contributed by early defects in protein sorting at the Golgi rather than from a retrieval defect alone. Our study supports previous ideas that UNC-16 is involved in multiple trafficking pathways. UNC-16 may achieve these functions via different downstream effector molecules that it can potentially scaffold, regulating different subsets of trafficking pathways. Our study shows that UNC-16 acts via downstream molecules LRK-1, AP-1 and AP-3 to control biogenesis of the SVP transport carriers in the cell body (Fig 8a). Previous biochemical studies in mammalian cells have indicated that LRRK2 was co-purified with both JIP3 and components of clathrin [31,62], suggesting that similar relationships may be conserved in mammals. We uncover a likely hierarchical series of processes regulated by these proteins, which occurs early on at the Golgi and post-Golgi compartments, for the sorting and regulation of SVP trafficking (Fig 8a).

Both, UNC-16 and LRK-1 proteins have been implicated to have roles at the synapse. UNC-16 has been shown to regulate RAB-5-mediated membrane trafficking and contribute to SV maturation at the synapses [34]. Lee *et*. *al*., 2010 and Piccoli *et*. *al*., 2011 have proposed presynaptic roles for LRRK2 where it regulates synaptic morphology and SV recycling dynamics respectively [62,63]. Thus, both UNC-16 and LRK-1 could have trafficking roles at the synapse in addition to or as a consequence of altered membrane composition arising at the Golgi.

## Materials and methods

### Strain maintenance

*C*. *elegans* strains were grown and maintained at 20°C on NGM plates seeded with the *E*. *coli* OP50 strain using standard methods [64]. L4 or 1-day adult animals were used for imaging in all cases. Strains used are listed in S4 Table. Some of the strains were provided by the CGC (https://cgc.umn.edu/acknowledging-the-cgc).

### Isolation and identification of *tb109*

The *unc-16* (*tb109*) allele was isolated from a behavioral suppressor screen carried out in *unc-104* (*e1265*) worms. This was a non-clonal screen of approximately 60,000 haploid genomes. Animals were mutagenized using 50 mM EMS for 4 h. F1 and F2 progenies were screened for improved locomotion. Suppressors were further identified by improved localization of GFP::RAB-3 in PLM neuron (Kumar *et al*., 2010). The *tb109* allele was separated from the background mutation and mapped to chromosome III and tested for non-complementation with *unc-16* (*e109*). The *tb109* phenotypes could be rescued by the expression of *P*_*unc-16*_::*UNC-16*::*GFP*. Sequencing revealed that allele *tb109* contains a point mutation in exon 9 leading to a stop codon at amino acid 423 (Arginine to opal as stop codon).

### Electron microscopy

Young adults of *unc-16* (*tb109*) and N2 were fixed for electron microscopy by high-pressure freezing (HPF) technique [65]. Serial sections were cut and the dorsal and ventral nerve cord regions were imaged using Gatan side mount camera on Tecnai G2 12 BioTwin electron microscope (FEI Company). The cross-sectional width of all the vesicles present in each section was measured using ImageJ [66].

### Immunostaining

Immunostaining was performed as described previously [16]. For double labeling of the SV proteins, sample was first incubated with mouse anti-RAB-3 (1:2000), followed by incubation with rabbit anti-SNT-1 (1:500) antibody. Appropriate secondary antibodies (1:350) (Alexa 488, Alexa568; Molecular probes) were added and incubated for two days. Images were captured using a Zeiss Axiovert inverted microscope. Images were processed using ImageJ [66].

### Immunoprecipitation

Samples were prepared by mechanical homogenization using homogenization buffer (15mM HEPES-NaOH pH7.4, 10mM KCl, 1.5mM MgCl2, 0.1mM EDTA, 0.5mM EGTA, 0.05mM sucrose and protease inhibitors (Roche)) followed by mild sonication at 4°C. Sample was then incubated with mouse monoclonal anti–Flag (1:50) (Biovision) or mouse monoclonal anti-GFP sera (1:10) (Genei, Merck) at 4°C for 4 h. The antigen-antibody complex was incubated with Protein-A agarose beads (Genei, Merck) for another 4 h. Antibodies used for Western blots to probe for UNC-16::GFP and LRK-1::Flag were rabbit anti-GFP (1:500–1000, Santacruz, Abcam) and mouse anti-flag (1:1000–2000, Sigma) respectively.

### Imaging and analysis

#### (i). Static imaging

L4 or 1-day adult worms were immobilized using 30mM Sodium Azide and mounted on 2–5% agarose pad. Images were acquired on an Olympus IX71 Epifluorescence Microscope with an Andor Camera, an Olympus IX73 Epi-fluorescence microscope with Evolution Camera or the Olympus IX83 with Perkin Elmer Ultraview Spinning Disc microscope and a Hamamatsu EMCCD camera or the Olympus Fluoview FV1000 confocal laser-scanning microscope. Imaging conditions vary from experiment to experiment.

#### (ii). Time lapse imaging

L4 worms were anesthetized in 5 mM Levamisole (Sigma-Aldrich) and mounted on 2% or 5% agarose pad. Time-lapse images were acquired in Olympus IX83 with Perkin Elmer Ultraview Spinning Disc microscope and a Hamamatsu EMCCD camera. Imaging conditions vary from experiment to experiment.

#### (iii). Analysis

Image analysis was done using ImageJ software [66].

#### Size analysis

Kymographs from a time-lapse image were made utilizing the “MultipleKymographs” ImageJ plugin. In a kymograph, straight vertical lines represent static particles whereas lines with a positive or negative slope represent moving particles. The length of the moving compartments was quantified by measuring the distance along the x-axis of the sloped line. This was done at regions not overlapping with stationary particles or other moving particles. Particles having length greater than 0.6μm was considered as longer compartments. We chose the 0.6μm cut-off for the following reasons: (i) The limit of resolution in our microscope is about 200nm leading to possible errors in length estimation in this size range, (ii) in wild type the majority of SVP carriers are between 0.4–0.6μm. Vesicles above 0.6μm in length are easy to measure accurately and very rare in wild type.

#### Co-migration analysis

Kymographs were made from identical regions of the movie in both colour channels utilizing the ImageJ plugin “MultipleKymographs”. The kymographs were then aligned and the number of sloped lines that overlapped was considered as co-migrating particles.

#### Quantification of the number of UNC-101 puncta (see Fig 6)

First a threshold for an image was set using the background intensity as cut-off value and then the number of puncta were quantified. To calculate the ratio of intensity, intensity measurements of a circle with fixed area (5x5 pixels) were taken from both puncta and the background regions.

#### Microtubule polarity by EBP-2 live imaging

Kymographs from a time-lapse image were made utilizing the “MultipleKymographs” ImageJ plugin. Number of EBP-2::GFP comets (anterograde and retrograde) were quantified from kymographs.

### Statistics

In all graphs, data are presented as mean values ± SEM. Statistical analysis was performed using GraphPad Prism 6 (GraphPad Software). Wherever possible, an independent t-test was used, or for multiple comparisons, one-way ANOVA followed by Dunett’s multiple comparison tests was done. For grouped datasets, two-way ANOVA, followed by Tukey’s multiple comparison tests was used. Differences were considered significant when P < 0.05 (*, P < 0.05; **, P < 0.01; ***, P < 0.001).

## Supporting information

S1 Fig UNC-16 regulates the trafficking of SVP as well as dendritic proteins. **(a)** Schematic diagram of UNC-16, with reported and newly identified (*tb109*) lesions of *unc-16* at the indicated positions. The predicted functional domains are colour-coded. **(b)** SNB-1::GFP is mis-localized into the dendritic compartment of amphid sensory neuron in two different alleles of *unc-16* (*e109* and *tb109*). The mis-localization is eliminated by an UNC-16 rescue construct (*tb109; kmEx1000*). **(c)** Dendritic protein ODR-10::GFP is mis-trafficked to the axon of the AWB neuron in *unc-16* (*tb109*) mutants. This defect in *unc-16* cannot be rescued by over-expression of LRK-1 (*unc-16*; *kmEx1180*). Red arrows indicate axonal localization. **(d)** The fraction of EBP-2::GFP comets moving in either anterograde or retrograde directions in both axon and dendrites in wild type and *unc-16* (*tb109*) are similar. This suggests that microtubule polarity is unchanged in *unc-16* (*tb109*) mutants. No. of animals examined = 5 per genotype; no. of EBP-2 comets quantified ≥ 60 **(e)** Double immunostaining of endogenous RAB-3 and SNT-1 in the sublateral neuronal process of wild type and *unc-16*. Merge panel shows the degree of co-localization between the two proteins. White arrows indicate an absence of co-localization. Scale bar represents 10 μm.
(TIF)

S2 Fig Golgi resident enzymes are mis-localized in *unc-16* mutants. **(a)** Quantitation from dual colour imaging and kymograph analysis shows Golgi enzyme ST::GFP is co-transported in the same compartment along with synaptic vesicle marker mCherry::RAB-3 into the PLM neuronal processes of *unc-16* (*tb109*) animals but not of *lrk-1* (*km17*) or *unc-101* (*m1*) animals. **(b)** Imaging of ST::GFP shows that the Golgi enzyme exits the cell body only to a low extent in *dhc-1* (*js319*) animals and not at all in *jnk-1* (*gk7*) animals. **(c)** Imaging of Man-II::GFP shows that it is mis-trafficked into dendrites in *unc-16* and *lrk-1* (*km17*) mutants. However, this accumulation depends on UNC-101 as indicated by *unc-101*(*m1*); *unc-16* (*tb109*) and *unc-101* (*m1*), *lrk-1* (*km17*) double mutants. Red arrows indicate dendritic tip accumulation. **(d)** Static images of neuronal cell bodies showing expression of Man-II::mCherry (PLM), ST::GFP (PLM), RUND-1::TagRFP (tail ganglia) and eGFP::RAB6.2 (ventral nerve cord) is shown. The number of puncta seen within each cell body has been quantified and shown as a fraction of total number of cell bodies examined. n ≥ 50 cell bodies. Scale Bar: In kymographs, horizontal scale bar represents 5 μm and vertical scale bar represent 40 sec. In image panel, scale bar represents 10 μm.
(TIF)

S3 Fig Distribution of length of SVP transport carriers seen in different genotypes. The graphs show a frequency distribution of the lengths measured of SVP transport carriers across different genotypes. **(a)** Comparison between wild type and two different mutant alleles of *unc-16* –*tb109* and *e109*. **(b)** Comparison between wild type and two different mutant alleles of *lrk-1* –*km17* and *km41*. **(c)** Comparison between wild type, *unc-16* (*tb109*) and LRK-1 overexpression in *unc-16* (*tb109*) animals. **(d)** Comparison between wild type, *unc-101* (*m1*) and LRK-1 overexpression in *unc-101*; *unc-16* animals. In each genotype, n ≥ 200 particles.
(TIF)

S4 Fig Interaction between UNC-16 and LRK-1. **(a) and (b)** show immunoprecipitation from animals overexpressing (a) LRK-1::FLAG and (b) UNC-16::GFP animals, where either LRK-1::FLAG or UNC- 6::GFP was pulled down and probed for the other protein. **(c)** Static images of LRK-1::Venus fluorescence in sensory and pharyngeal neurons shows that LRK-1::Venus is mis-localized to dendrites in *unc-16* (*tb109*) mutants. **(d)** Quantitation and comparison of punctate distribution of LRK-1::Venus in neuronal cell bodies between wild type and *unc-16* (*tb109*) animals. n ≥ 25 cell bodies **(e)** Static images showing punctate distribution of UNC-16::GFP in the ventral nerve cord in wild type and *lrk-1* (*km17*) animals. Quantitation and comparison of puncta distribution suggests that UNC-16 localization is not affected in *lrk-1* mutants. n ≥ 25 cell bodies **(f)** Anterograde flux measurements and comparison between wild type, *unc-16* (*tb109*), *unc-104* (*e1265*); *unc-16* (*tb109*), *unc-116* (*e2310*); *unc-104* (*e1265*); *unc-16* (*tb109*) mutants indicates that flux in *unc-16* decreases in absence of Kinesin-3 and Kinesin-1. This further suggests that the RAB-3 containing transport carriers depend on multiple motors. n ≥ 10 animals.
(TIF)

S1 Table Quantitation of dendritic mis-localization of synaptic vesicle marker (SNB-1::GFP) and Golgi-resident enzyme (Man-II::GFP) in amphid sensory neurons in various mutant backgrounds. (XLSX)

S2 Table Presence of large synaptic vesicle protein transport carriers in different mutant backgrounds, visualized using two independent synaptic vesicle markers (GFP::RAB-3 and SNB-1::GFP). (XLSX)

S3 Table Comparison between phenotypes seen in *unc-16*, *lrk-1*, *lrk-1; unc-16* and *unc-101* mutants. (XLSX)

S4 Table List of strains used in this study. (XLSX)

S1 Movie Golgi enzyme Mannosidase-II remains restricted to the cell body in wild type animals. Dual colour live imaging of the PLM neuronal process was done (3fps for 3 min at 100 X) using wild type L4-animals expressing GFP::RAB-3 and Man-II::mCherry.
(AVI)

S2 Movie Golgi enzyme Mannosidase-II is co-transported along with SVP RAB-3 in the neuronal process of *unc-16* animals. Dual colour live imaging of the PLM neuronal process was done (3fps for 3 min at 100 X) using *unc-16* (*tb109*) L4-animals expressing GFP::RAB-3 and Man-II::mCherry.
(AVI)

S3 Movie Size of RAB-3-tagged transport carriers in wild type animals. Live imaging of the PLM neuronal process was done (5fps for 3 min at 100 X) using wild type L4-animals expressing GFP::RAB-3 shows no large compartments.
(AVI)

S4 Movie RAB-3 tagged large compartments are seen in PLM neurons of *unc-16* mutants. Live imaging of the PLM neuronal process was done (5fps for 3 min at 100 X) using *unc-16* (*tb109*) L4-animals expressing GFP::RAB-3 shows large compartments.
(AVI)

S5 Movie SNB-1 tagged large compartments are seen in ASI neuronal process of *unc-16* mutants. Live imaging of the ASI neuronal process was done using *unc-16* (*tb109*) L4-animals expressing SNB-1::GFP shows large tubular compartment in the dendritic process.
(AVI)

S6 Movie RAB-3 tagged large compartments are seen in PLM neurons of *unc-101* mutants. Live imaging of the PLM neuronal process was done (5fps for 3 min at 100 X) using *unc-101* (*m1*) L4-animals expressing GFP::RAB-3 shows large compartments.
(AVI)

S7 Movie RAB-3 tagged large compartments are seen in motor neurons of *unc-101* mutants. Live imaging of the motor neurons was done (5fps for 3 min at 100 X) using *unc-101* (*m1*) L4-animals expressing GFP::RAB-3 shows large compartments.
(AVI)
