# Supplementary figures and images for: UNC-16/JIP3 regulates early events in synaptic vesicle protein trafficking via LRK-1/LRRK2 and AP complexes
Source: PLoS Genet. 2017 Nov 16;13(11):e1007100. doi: 10.1371/journal.pgen.1007100 (PMC5716593; doi:10.1371/journal.pgen.1007100)

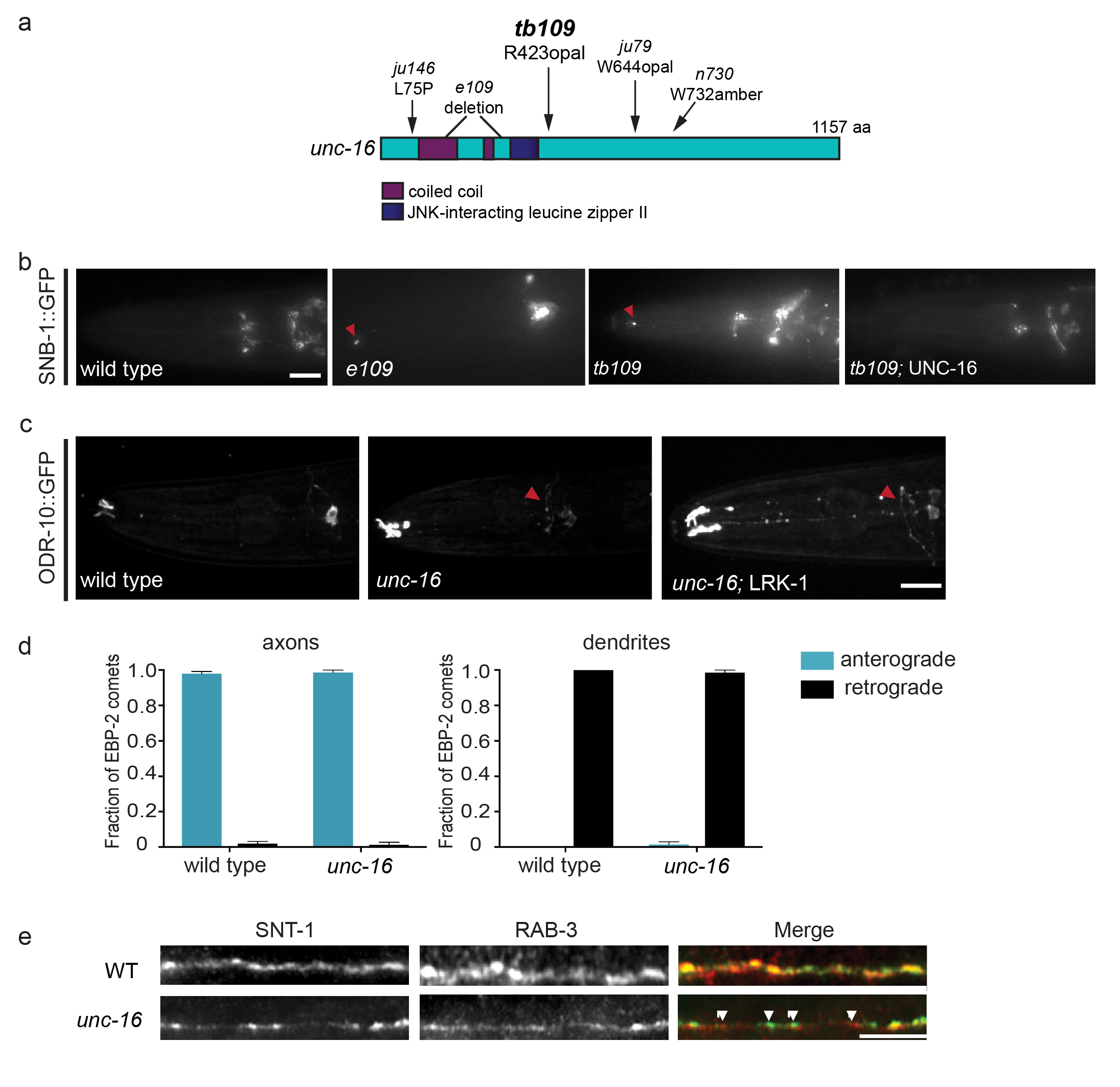

Supplement: S1 Fig — (a) Schematic diagram of UNC-16, with reported and newly identified (tb109) lesions of unc-16 at the indicated positions. The predicted functional domains are colour-coded. (b) SNB-1::GFP is mis-localized into the dendritic compartment of amphid sensory neuron in two different alleles of unc-16 (e109 and tb109). The mis-localization is eliminated by an UNC-16 rescue construct (tb109; kmEx1000). (c) Dendritic protein ODR-10::GFP is mis-trafficked to the axon of the AWB neuron in unc-16 (tb109) mutants. This defect in unc-16 cannot be rescued by over-expression of LRK-1 (unc-16; kmEx1180). Red arrows indicate axonal localization. (d) The fraction of EBP-2::GFP comets moving in either anterograde or retrograde directions in both axon and dendrites in wild type and unc-16 (tb109) are similar. This suggests that microtubule polarity is unchanged in unc-16 (tb109) mutants. No. of animals examined = 5 per genotype; no. of EBP-2 comets quantified ≥ 60 (e) Double immunostaining of endogenous RAB-3 and SNT-1 in the sublateral neuronal process of wild type and unc-16. Merge panel shows the degree of co-localization between the two proteins. White arrows indicate an absence of co-localization. Scale bar represents 10 μm. (TIF) [file pgen.1007100.s001.tif]

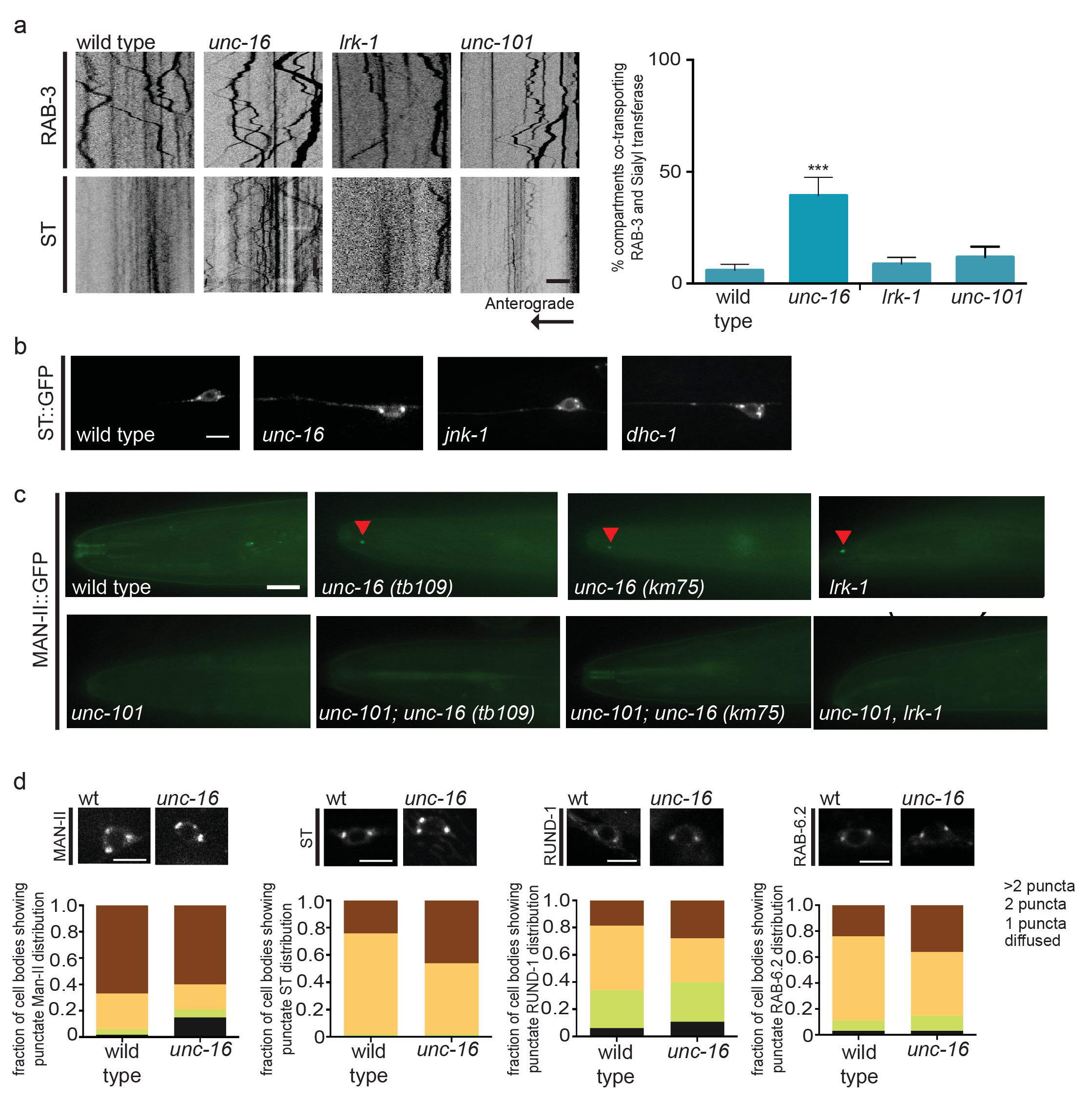

Supplement: S2 Fig — (a) Quantitation from dual colour imaging and kymograph analysis shows Golgi enzyme ST::GFP is co-transported in the same compartment along with synaptic vesicle marker mCherry::RAB-3 into the PLM neuronal processes of unc-16 (tb109) animals but not of lrk-1 (km17) or unc-101 (m1) animals. (b) Imaging of ST::GFP shows that the Golgi enzyme exits the cell body only to a low extent in dhc-1 (js319) animals and not at all in jnk-1 (gk7) animals. (c) Imaging of Man-II::GFP shows that it is mis-trafficked into dendrites in unc-16 and lrk-1 (km17) mutants. However, this accumulation depends on UNC-101 as indicated by unc-101(m1); unc-16 (tb109) and unc-101 (m1), lrk-1 (km17) double mutants. Red arrows indicate dendritic tip accumulation. (d) Static images of neuronal cell bodies showing expression of Man-II::mCherry (PLM), ST::GFP (PLM), RUND-1::TagRFP (tail ganglia) and eGFP::RAB6.2 (ventral nerve cord) is shown. The number of puncta seen within each cell body has been quantified and shown as a fraction of total number of cell bodies examined. n ≥ 50 cell bodies. Scale Bar: In kymographs, horizontal scale bar represents 5 μm and vertical scale bar represent 40 sec. In image panel, scale bar represents 10 μm. (TIF) [file pgen.1007100.s002.tif]

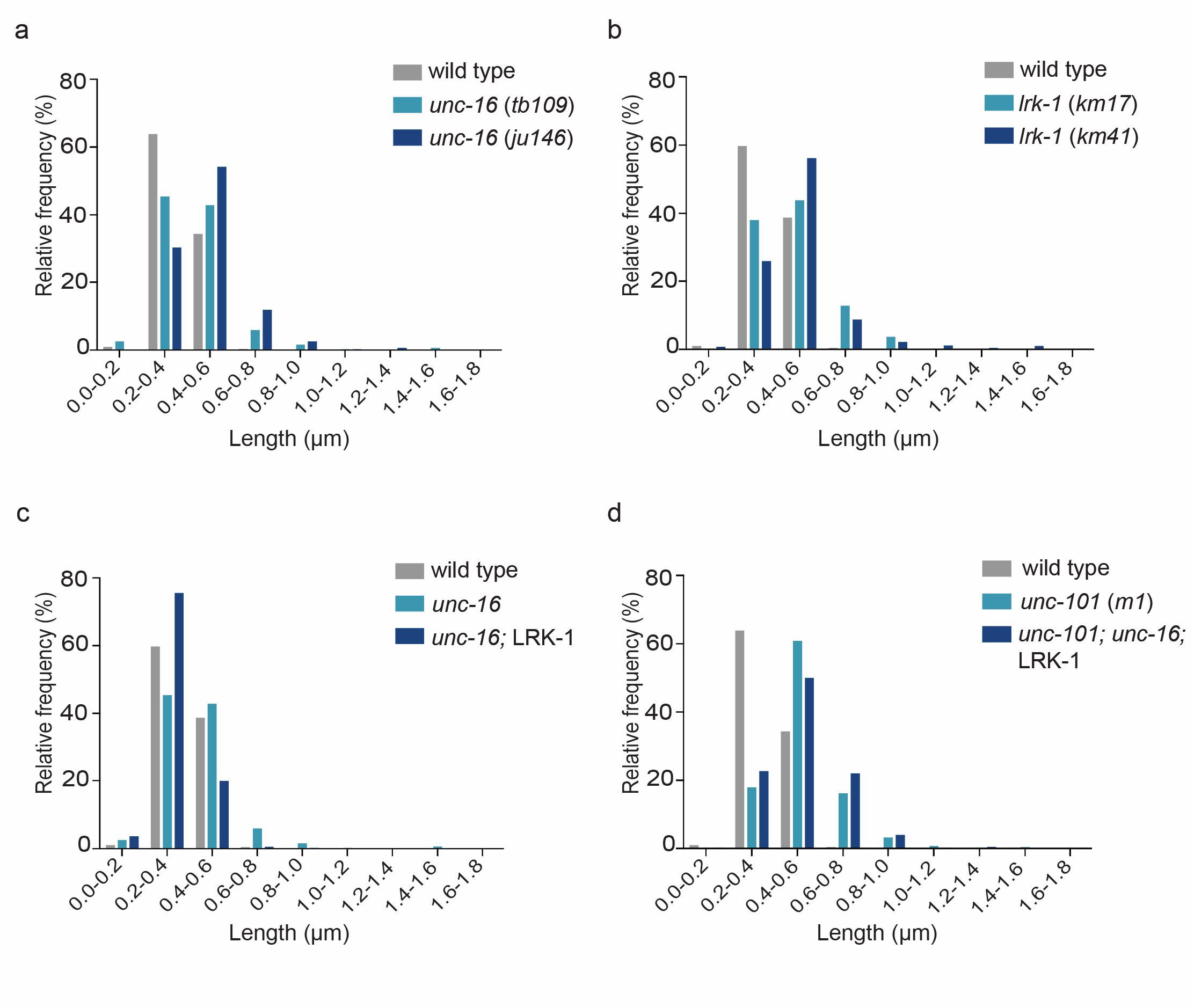

Supplement: S3 Fig — The graphs show a frequency distribution of the lengths measured of SVP transport carriers across different genotypes. (a) Comparison between wild type and two different mutant alleles of unc-16 –tb109 and e109. (b) Comparison between wild type and two different mutant alleles of lrk-1 –km17 and km41. (c) Comparison between wild type, unc-16 (tb109) and LRK-1 overexpression in unc-16 (tb109) animals. (d) Comparison between wild type, unc-101 (m1) and LRK-1 overexpression in unc-101; unc-16 animals. In each genotype, n ≥ 200 particles. (TIF) [file pgen.1007100.s003.tif]

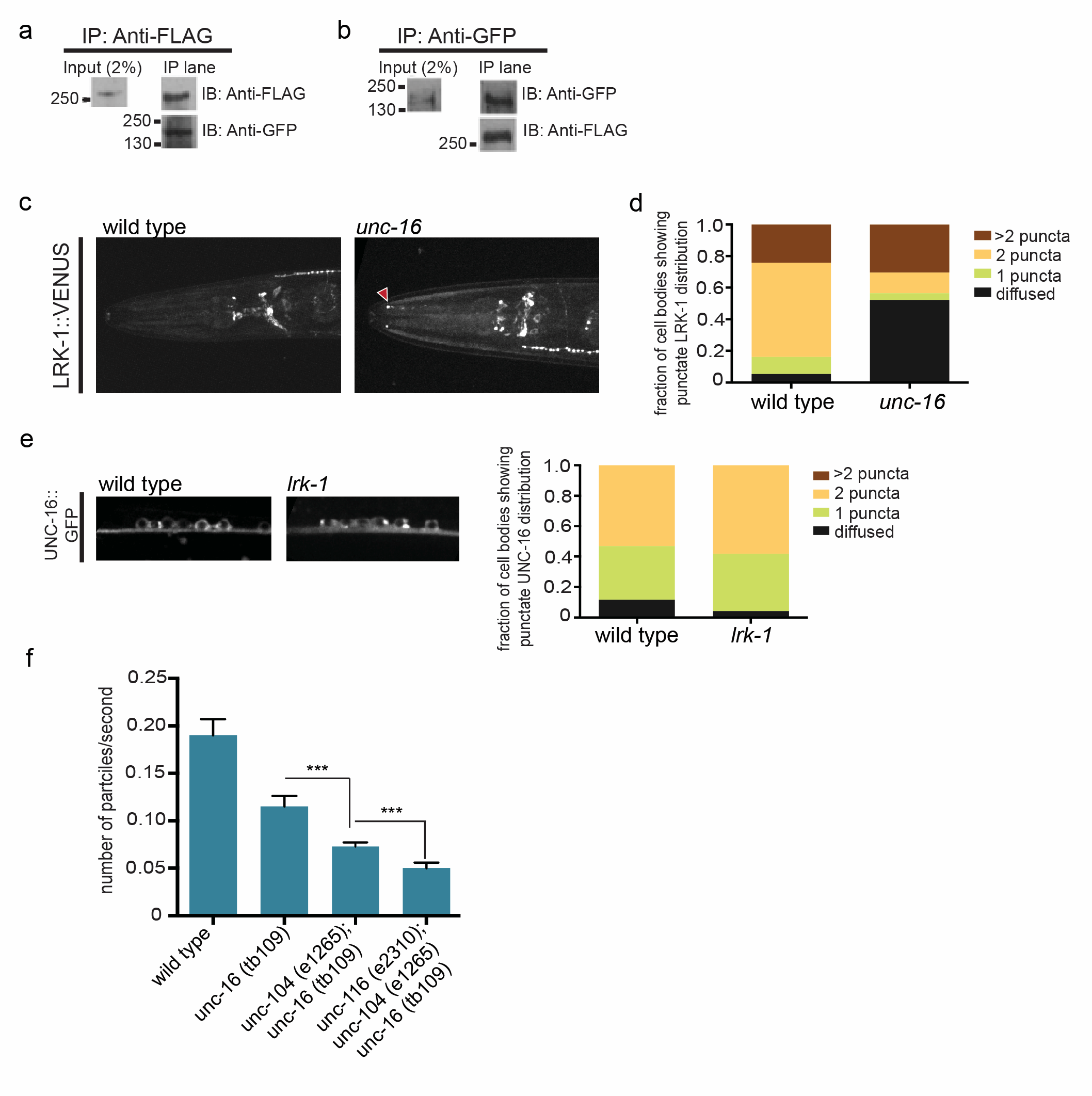

Supplement: S4 Fig — (a) and (b) show immunoprecipitation from animals overexpressing (a) LRK-1::FLAG and (b) UNC-16::GFP animals, where either LRK-1::FLAG or UNC- 6::GFP was pulled down and probed for the other protein. (c) Static images of LRK-1::Venus fluorescence in sensory and pharyngeal neurons shows that LRK-1::Venus is mis-localized to dendrites in unc-16 (tb109) mutants. (d) Quantitation and comparison of punctate distribution of LRK-1::Venus in neuronal cell bodies between wild type and unc-16 (tb109) animals. n ≥ 25 cell bodies (e) Static images showing punctate distribution of UNC-16::GFP in the ventral nerve cord in wild type and lrk-1 (km17) animals. Quantitation and comparison of puncta distribution suggests that UNC-16 localization is not affected in lrk-1 mutants. n ≥ 25 cell bodies (f) Anterograde flux measurements and comparison between wild type, unc-16 (tb109), unc-104 (e1265); unc-16 (tb109), unc-116 (e2310); unc-104 (e1265); unc-16 (tb109) mutants indicates that flux in unc-16 decreases in absence of Kinesin-3 and Kinesin-1. This further suggests that the RAB-3 containing transport carriers depend on multiple motors. n ≥ 10 animals. (TIF) [file pgen.1007100.s004.tif]
